# Recent advancements in the synthesis of *Veratrum* alkaloids

**DOI:** 10.3762/bjoc.21.206

**Published:** 2025-12-10

**Authors:** Morwenna Mögel, David Berger, Philipp Heretsch

**Affiliations:** 1 Institut für Organische Chemie, Leibniz Universität Hannover, Schneiderberg 1b, 30167 Hannover, Germany

**Keywords:** alkaloid synthesis, convergent synthesis, total synthesis, *Veratrum* alkaloids

## Abstract

The *Veratrum* alkaloids constitute a class of natural products with particularly intricate polycyclic frameworks and dense stereochemistry and, thus, have stood long as benchmarks in chemical synthesis. Recently, these steroid alkaloids gained popularity as challenging targets in total synthesis, with a clear shift toward convergent strategies. Not only do these syntheses feature rapid assembly of their challenging cores through modular and strategic bond connections, but they also give a reflection on the advancement of modern synthetic methods and retrosynthetic logic. This review will cover recent syntheses, highlighting the convergence of modern strategic disconnections, stereocontrol, and late-stage functionalization for rapid access to these exceptional alkaloids, while also showcasing the evolution of the art of synthesis and its ability in meeting the demands posed by molecular complexity.

## Introduction

**Scheme 1:**
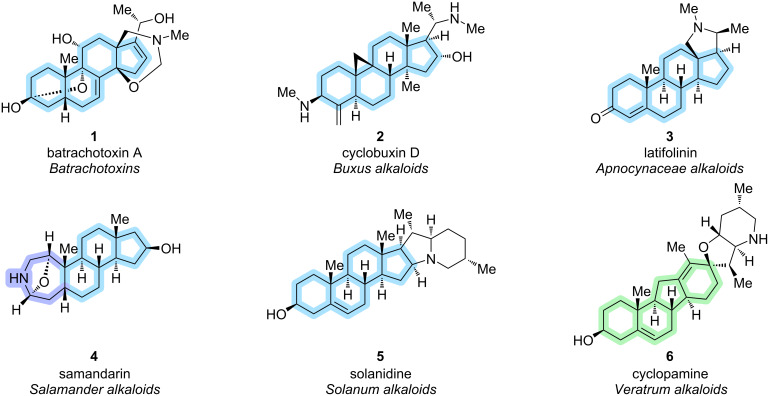
Representatives of steroid alkaloid classes. Marked in blue is the steroidal cholestane framework, rearrangement of ring A in samandarin (**4**, bottom left) in purple, rearranged *abeo*-motif of *Veratrum* alkaloids (bottom right) in green [1].

*Veratrum* alkaloids belong to the class of steroid alkaloids and are pseudoalkaloids, as they emerge biogenetically from steroidal biosynthesis via cholesterol and not, as usual for alkaloids, from amino acids [1]. For synthetic chemists, this combines challenges for steroid and alkaloid total synthesis and poses unique obstacles for synthetic strategies [2]. The steroid alkaloids include *Solanum*, *Veratrum*, *Apocynaceae*, and *Buxus* alkaloids in the kingdom of plants, and salamander alkaloids and batrachotoxins, which include one of the most toxic compounds known, in the animal kingdom [1]. Looking at common representatives (Scheme 1), the steroidal C27-cholestane backbone is easily recognizable, except for a rearranged 14(13→12)*abeo*-cholestane in the *Veratrum* alkaloids. This C-*nor*-D-*homo*-framework represents a unique distinction from the other classes, requiring different and creative approaches for its synthesis. The skeleton is marked in blue for batrachotoxin A (**1**) [3], cyclobuxin D (**2**) [4], latifolinin (**3**) and solanidine (**5**) [5]. In samandarin (**4**) [6], the A-ring consists of a seven-membered nitrogen-containing ring (marked in purple), but the BCD-framework remains unchanged. The representative *Veratrum* alkaloid, cyclopamine (**6**) [7], displays the C-*nor*-D-*homo* framework (marked in green).

**Scheme 2:**
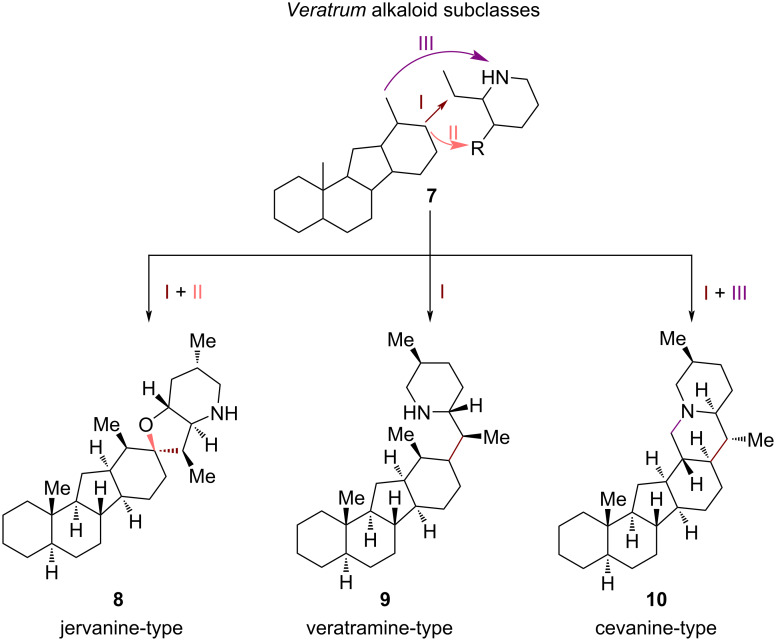
Subclasses of *Veratrum* alkaloids: jervanine, veratramine and cevanine-type [8].

*Veratrum* alkaloids can be further divided into three subclasses: jervanine, veratramine, and cevanine-type *Veratrum* alkaloids (Scheme 2) [8]. Main differentiation of these structural motifs involves the connectivity of the C-*nor*-D-*homo* skeleton to an EF-ring system; jervanine-type alkaloids consist of a hexacyclic framework with a spirofused tetrahydrofuran E-ring and a fused piperidine F-ring (connectivity I + II, Scheme 2). Veratramine-type *Veratrum* alkaloids resemble the jervanine-type subclass with respect to the piperidine motif but lack the E-ring (connectivity I, Scheme 2). Most congeners of this subclass also include an aromatic D-ring, but also alkaloids with non-aromatic D-rings belong to this group. Cevanine-type alkaloids are the largest subgroup in the *Veratrum* class. In comparison to the veratramine subclass, an additional E-ring is formed via connection of an sp^3^-center at the D-ring to the nitrogen atom of the piperidine F-ring, thus, forming a quinolizidine EF-moiety (connectivity I + III, Scheme 2). Members of this group are often highly oxidized, featuring multiple hydroxy groups embedded in A–E rings, thus, adding to their complexity in total synthesis efforts [9].

**Scheme 3:**
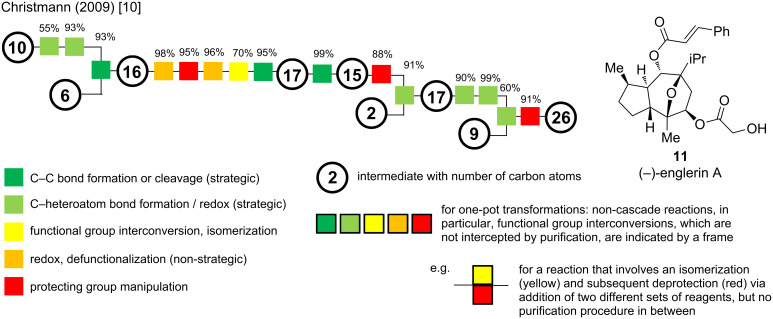
Flow chart presentation of the synthesis of (−)-englerin A developed by the Christmann group [10].

This review will cover synthetic approaches from the last decades, showcasing the evolution of *Veratrum* alkaloids synthesis and by that, providing an impression of the evolution of synthetic methods in general. For a visual comparison, we decided to implement flow charts with colored boxes and reaction classifications as suggested by Schwan and Christmann [10]. In their publication, reaction steps are ranked by a color scheme resembling a traffic light, depending on their strategic value in the synthesis: two shades of green for strategic connections, yellow for neutral, e.g., functional group interconversion and isomerization, orange for non-strategic reactions, and red for protecting group (PG) manipulations (Scheme 3). Protecting group manipulations refer to any protection or deprotection of a functional group in the sequence to temporarily mask that specific group and its reactivity. The latter are considered undesirable transformations, since they do not contribute to advancing the complexity of the synthetic intermediate toward the target and should therefore be avoided, or at least minimized in use. For intermediates, circles are added with the number of carbon atoms (carbons included in the skeleton, carbon atoms of protecting groups are not counted). We decided to deviate from the originally proposed step-count and will stick to the definition of a reaction step being the sum of all transformation carried out in one flask terminated by a purification [11]. Nevertheless, to point out reaction telescoping (i.e., performing several reactions in one flask by sequentially adding reagents, including solvent-swaps) we opted for a visual representation indicated by black frames around the boxes, color-coding each transformation in this one-pot procedure. Cascade reactions, as well as reactions, where a single set of reaction conditions and reagents transform two or more groups simultaneously (e.g., global deprotections) and reactions, that occur upon work-up, are still regarded as one step and will not be shown separately. For an example for this color coding, the (−)-englerin A (**11**) synthesis of the Christmann group is displayed in Scheme 3, together with the symbol explanation mentioned above [12].

This visual aid helps identifying the convergency and efficiency of synthetic routes, as well as showcasing step- and redox-economy. This representation will be highly valuable for comparing the presented synthetic routes at the end of each subclass.

## Review

### Synthesis of *Veratrum* alkaloids

The biological activities of the *Veratrum* alkaloids have been widely studied, but their synthesis remained a major challenge for synthetic chemists for decades [13]. The first syntheses were reported in the 1960s for jervine (**12**, Masamune, 1967) [14], veratramine (**13**, Johnson, 1967) [15], and verarine (**14**, Kutney, 1968) [16–19]. A decade later, the Kutney group published a semisynthetic approach to verticine (**15**) [20], which remained the only reported synthesis of a cevanine-type alkaloid for over 40 years.

In 2009, the Giannis group reported the first synthesis of cyclopamine (**6**), a jervanine-type alkaloid, via a biomimetic semisynthetic approach, breaking the ice for further studies in this isosteroidal alkaloid class [21]. Stork followed in 2017 with a synthesis to 4-methylene-germine (**17**), almost completing the total synthesis of germine (**20**) before his passing [22].

**Scheme 4:**
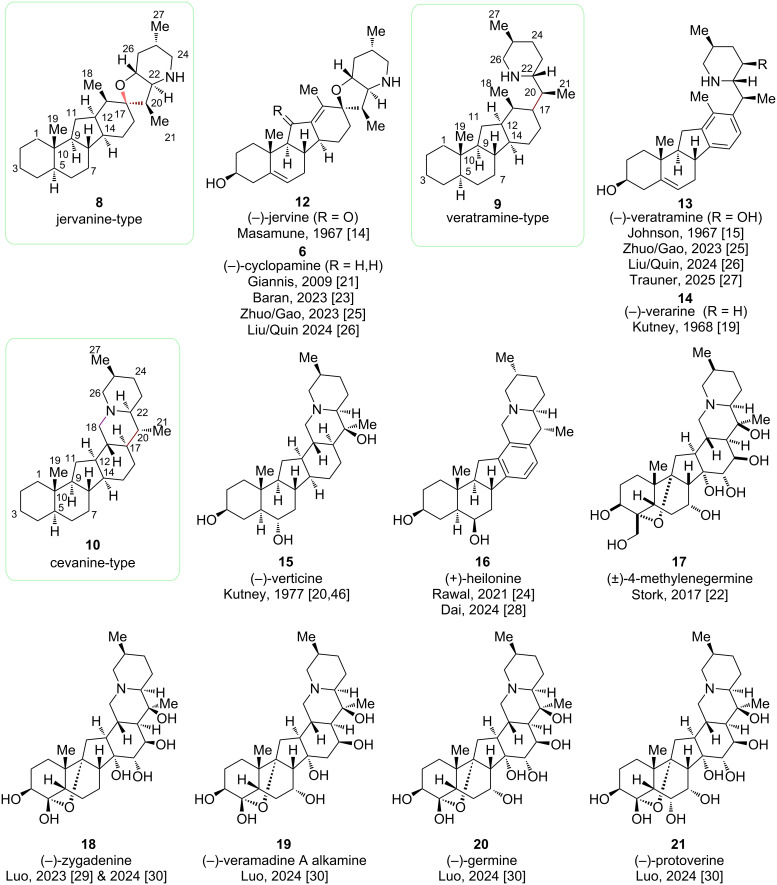
Structures and year of synthesis of the three types of *Veratrum* alkaloids reported in the literature [14–15,19–30].

The real renaissance in the total synthesis of *Veratrum* alkaloids started in the 2020s, rung in by the Baran group in 2023 and their synthesis of cyclopamine (**6**) [23], as well as with the total synthesis of heilonine (**16**) by the Rawal group in 2021 [24]. Since then, cyclopamine (**6**) and veratramine (**13**) have been revisited as targets by numerous groups and moreover, several highly oxidized cevanine-type *Veratrum* alkaloids have been successfully tackled (Scheme 4) [25–30].

### Synthesis in the jervanine subclass

In the jervanine class of *Veratrum* alkaloids, two structures have been revisited for semisynthesis and total synthesis approaches: cyclopamine (**6**) and jervine (**12**). In comparison, jervine possesses an additional oxo group at C11 in the C-ring.

#### Cyclopamine

The most prominent *Veratrum* alkaloid – cyclopamine – also has one of the most gruesome discovery stories [31–32]. In the 1950s, sheepherders in Idaho, USA, alarmed authorities after several cases of newborn lambs born one-eyed were reported [33]. This unsettling deformity with their single eye placed in the middle of the forehead gave them a cyclops-like appearance. Investigations surfaced that pregnant sheep had ingested *Veratrum californicum* (corn lily), and cyclopamine (**6**) was reported in 1957 as the culprit [34]. Later studies confirmed its mode of action by inhibition of the hedgehog signaling pathway, which plays a critical role in the differentiation and symmetry in the development of embryos [35].

We will have a further look into four different approaches to synthesize this natural product. The first synthesis was reported by the Giannis group in 2009 starting from dehydroepiandrosterone via a biomimetic rearrangement [21]. We will later discover and compare the improvements in the semisynthesis reported by Liu and Qin, which relies on a similar skeletal transformation toward (−)-cyclopamine and (−)-veratramine [26].

**Scheme 5:**
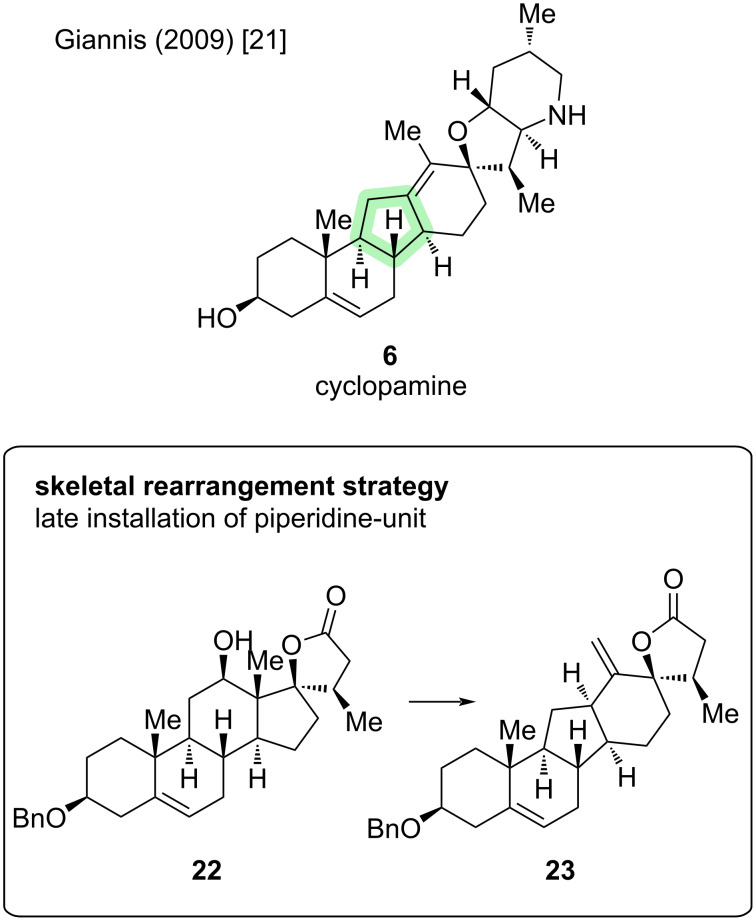
Key step in the synthesis of cyclopamine (**6**) by the Giannis group [21].

Key step of the first semisynthesis of (−)-cyclopamine (**6**) is a Wagner–Meerwein-type rearrangement of androstane-framework **22** toward the C-*nor*-D-*homo* skeleton in **23** (Scheme 5) [21].

**Scheme 6:**
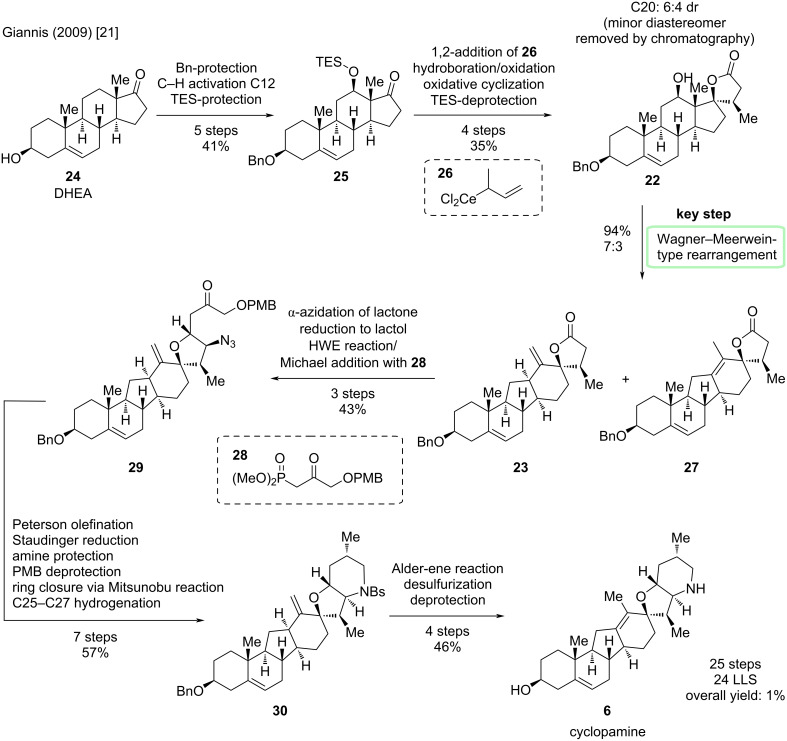
Overview of the semisynthesis of cyclopamine (**6**) reported by the Giannis group in 2009 [21].

The synthesis starts from commercially available dehydroepiandrosterone (DHEA, **24**) by protection of the free hydroxy group in 3-position, a copper-mediated C–H activation of position 12 and subsequent protection of the introduced hydroxy moiety to give compound **25** (Scheme 6). Installation of the lactone moiety in **22** was carried out in a three-step sequence via a 1,2-addition of organocerium reagent **26** to the ketone, hydroboration/oxidation, and an oxidative cyclization protocol. The diastereomers were separable by column chromatography, leading to this faster and more scalable procedure to be performed preferentially over a six-step diastereoselective sequence. After silyl ether deprotection, the Wagner–Meerwein-type rearrangement was carried out with trifluoromethanesulfonic anhydride leading to a mixture of regioisomers **23** and **27**, with **23** being convertible to **27** by addition of DBU. The A–E-rings have, thus, been installed with correct stereoconfiguration, therefore the last steps of the sequence focused on the introduction of the F-ring and isomerization of the *exo*-methylene motif in the D-ring. First, the nitrogen was introduced via an α-azidation of the lactone, which itself was then reduced to a lactol, enabling a Horner–Wadsworth–Emmons (HWE) reaction with phosphonate **28** to provide **29**. Peterson olefination installed another *exo*-methylene (in the future F-ring), which would later be reduced to the methyl group at C25. Staudinger reduction and two protecting group manipulations set the stage for the formation of the piperidine (F-ring) through a Mitsunobu reaction. Hydrogenation employing Wilkinson’s catalyst gave the protected sulfonamide **30**. For the isomerization of the *exo*-double bond, an Alder-ene reaction with *N*-sulfinylbenzenesulfonamide and subsequent desulfurization was the way of choice. Finally, both remaining protecting groups were removed to furnish cyclopamine (**6**). In total, **6** was prepared in 24 steps in the longest linear sequence (LLS) [originally counted as 20 steps, but the rules for step-counting highlighted above lead to this recalculation for reasons of comparability].

**Scheme 7:**
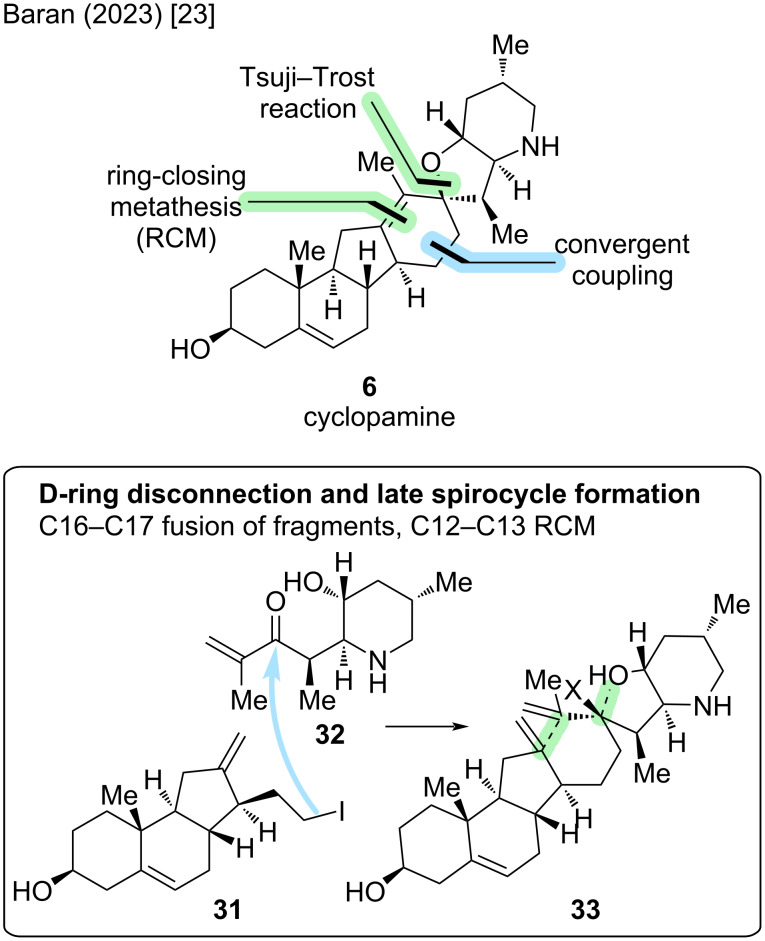
Key steps in the synthesis of cyclopamine (**6**) by the Baran group [23].

This synthesis remained the only reported synthesis of cyclopamine (**6**) for more than a decade, until the Baran group disclosed a total synthesis in 2023 [23]. Their synthesis relies on a retrosynthetic cut through the D-ring, enabling a convergent coupling of two similarly complex fragments between C15–C16. Key disconnection steps include a Tsuji–Trost reaction to form the spirocyclic E-ring, and a ring-closing metathesis for closing the D-ring (Scheme 7).

**Scheme 8:**
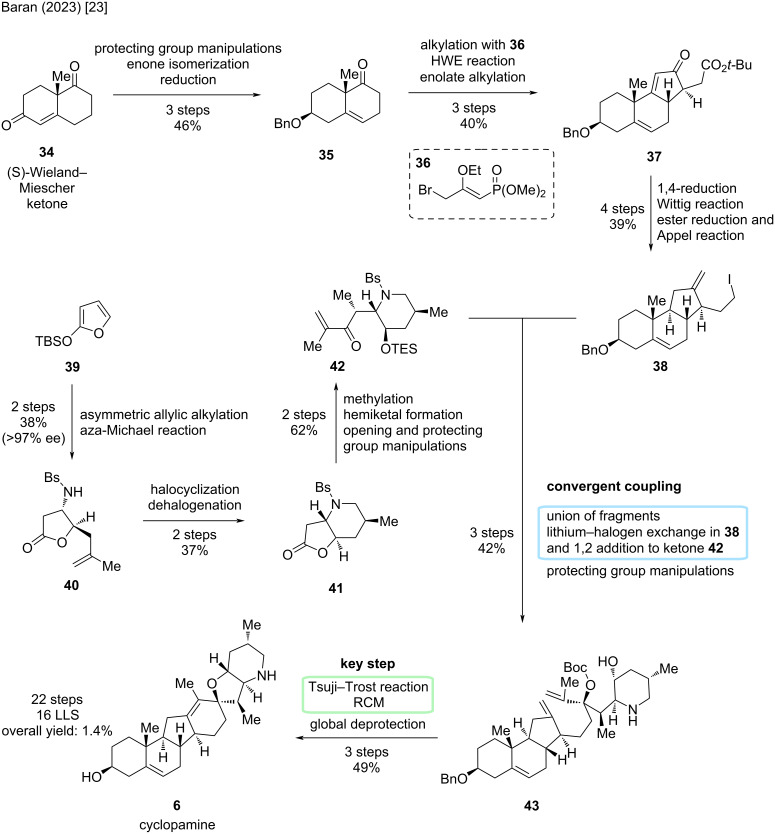
Overview of the total synthesis of cyclopamine (**6**) by the Baran group in 2023 [23].

For the left-hand fragment including the A-, B-, and C-ring, the synthesis started from commercially available (*S*)-Wieland–Miescher ketone (**34**), which can also be synthesized via a three-step sequence (Scheme 8). To access **35**, several protecting group manipulations were carried out, the double bond isomerized to position C5–C6, and a reduction was performed, all in an optimized three-step, scalable sequence. Alkylation with phosphonate reagent **36** and subsequent Horner–Wadsworth–Emmons (HWE) reaction formed ring C, followed by an enolate alkylation to forge substituted cyclopentenone motif **37**. The authors report the reaction to favor the desired diastereomer and could further enrich the desired isomer by recrystallization. Final steps for this fragment included a 1,4-reduction of the enone motif via a copper hydride species, a Wittig reaction to install the *exo*-methylene group and an ester reduction/Appel reaction sequence to convert the ester moiety to an iodide leaving group.

The right-hand fragment (E/F-ring) was synthesized from *tert*-butylsiloxyfuran **39**. Commencing via an asymmetric allylic alkylation and an aza-Michael reaction, butanolide **40** was obtained in 38% yield and an enantiomeric excess of greater than 97%. A halocyclization and subsequent dehalogenation was performed to arrive at fused compound **41**. Notably, similar compounds had been synthesized before, but the Baran group provided a faster access to this molecule. Methylation of **41** by enolate alkylation was diasteroselective. In the next steps, addition of isopropenyllithium to the lactone moiety led to a hemiketal, which was then opened and a protecting group on the hydroxy group was installed, furnishing fragment **42**. Key convergent coupling of this total synthesis was performed by lithium–halogen exchange in the ABC-fragment **38** followed by 1,2-addition to the ketone moiety in the F-ring fragment **42**. Protection group manipulations allowed for the union of both fragments to advanced intermediate **43** in three steps in 42%, setting the stage for the key cyclization reactions in this sequence. First, an alkoxide Tsuji–Trost reaction forged the spirocyclic E-ring, then a ring-closing metathesis closed the D-ring. Finally, global deprotection furnished cyclopamine (**6**) in 49% over three steps. In conclusion, the Baran group achieved a convergent synthesis of cyclopamine in 16 steps in the LLS (total steps: 22, overall yield 1.4%) and provided the first total synthesis of this molecule (in comparison to the Giannis group, who achieved the first synthesis of cyclopamine in a semisynthetic fashion).

**Scheme 9:**
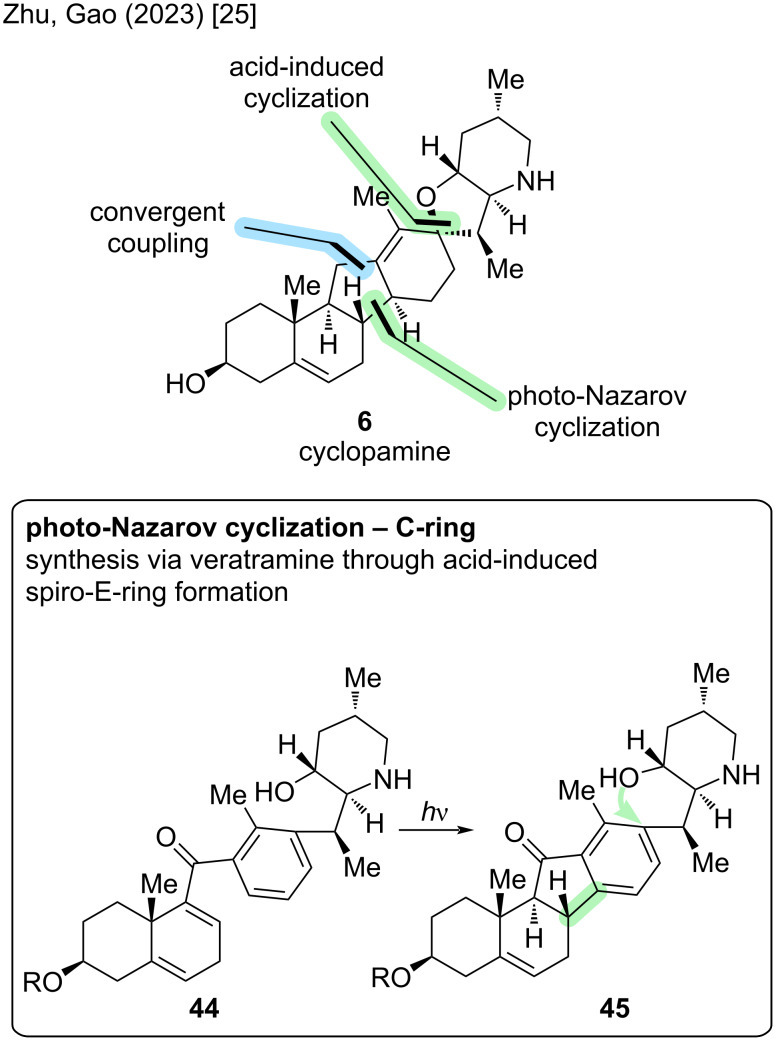
Key steps in the synthesis of cyclopamine (**6**) by the Zhu/Gao group [25].

We already discussed a possible disconnection through the D-ring and late-stage formation of the spirocyclic moiety. Another disconnection was provided by the group of Zhu/Gao in 2023 (Scheme 9) [25]. The major retrosynthetic cut proceeds through the C-ring, while also including a late-stage spirocyclization. The convergent coupling occurs between C11 and C12, while a ketone moiety is present at C11. Compound **44** could then be subjected to a Nazarov cyclization, which was performed in a photochemical fashion, to close ring C. Further reduction and hydrogenation in ring D and an acid-induced spirocyclization of Nazarov-product **45** concluded the synthesis of cyclopamine. Notably, this total synthesis of cyclopamine (**6**) proceeded through veratramine (**13**), before interconverting veratramine into cyclopamine. Therefore, the Zhu/Gao groups strategy provides access to both natural compounds; the synthesis will later also be discussed in the veratramine paragraph.

**Scheme 10:**
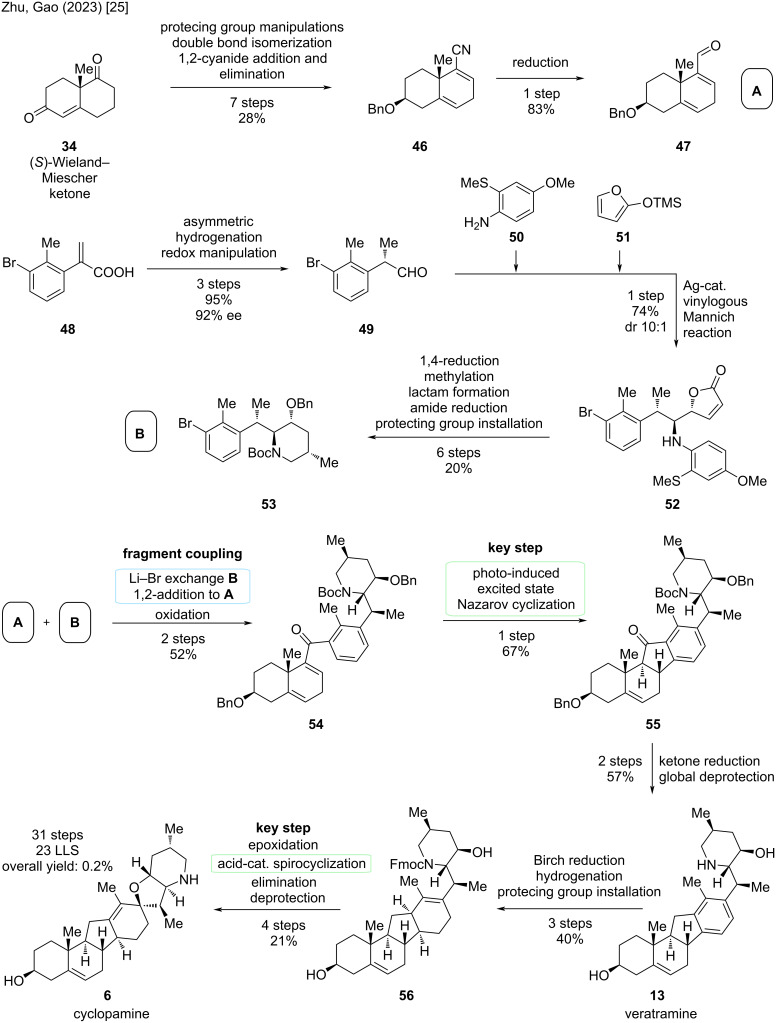
Overview of the total synthesis of cyclopamine (**6**) by the group of Zhao/Gao in 2023 [25].

For the synthesis of the left-hand fragment, the starting material was again the (*S*)-Wieland–Miescher ketone (**34**). This was transformed to nitrile **46** via a seven-step sequence consisting of protecting group manipulations, a double bond isomerization to position C5–C6, as well as a 1,2-cyanide addition, and subsequent elimination (Scheme 10). The cyanide moiety was introduced as a precursor to an aldehyde, which was obtained by reduction of **46** to **47**.

For the right-hand fragment (ring D, E, and F), an asymmetric hydrogenation of α-substituted acrylic acid **48** was performed, followed by redox manipulations to give aldehyde **49** over 3 steps in 95% and an enantiomeric excess of 92%. This asymmetric hydrogenation included a novel iridium catalyst featuring a chiral SpiroBAP (spiro bidentate aminophosphorane) ligand, which has been developed previously by this group and was now successfully applied in this synthesis [36]. A silver-catalyzed, enantioselective, vinylogous Mannich reaction of the in situ-generated aldimine condensation product of **49** and amine **50** with trimethylsiloxyfuran **51** generated secondary amine **52** in a diastereomeric ratio of 10:1. To close the piperidine, which is to become the F-ring, six steps were carried out: 1,4-reduction of the butenolide moiety, selective α-methylation, formation of a lactam, reduction of the former to an amine, and installation of two different protecting groups. Aryl bromide **53** (fragment B) was then ready for coupling.

Lithium–halogen exchange in fragment B **53** and subsequent 1,2-addition to fragment A **47** resulted in a hydroxy group, which was oxidized to furnish Nazarov precursor **54** in 52% yield. Key step of this sequence was the photoinduced excited-state Nazarov reaction, which involved irradiation of **54** in dichloroethane at 366 nm for 30 min at room temperature. Remarkably, this method had been developed by the group before [37] and successfully installed a cyclopentenone C-ring in 67% yield with the correct stereoconfiguration at C9 and C10, simultaneously. Ketone reduction and global deprotection provided veratramine (**13**) in two steps. For the transformation toward cyclopamine (**6**), hydrogenation of ring D and spirocyclization were to be executed. The next three steps included a Birch reduction, selective hydrogenation with PtO_2_, and protecting group installation to give **56** with the double bond at C13–C17, which was crucial for the following spirocyclization. This double bond was epoxidized selectively, and, through acid-catalyzed cyclization, the spiro-E-ring was fused. Elimination of the alcohol moiety at C13 installed the double bond in the correct position at C12–C13. Final Fmoc-deprotection furnished cyclopamine (**6**). In summary, the Zhu/Gao group disclosed the total synthesis of cyclopamine in 23 steps in the LLS, with a total step count of 31 (overall yield 0.2%). Albeit being significantly longer in the sequence than the reported synthesis of the Baran group [23], this approach provides an entirely different strategy and also displays new methodologies (asymmetric hydrogenation, Nazarov cyclization), while enabling a synthesis through veratramine (**13**).

Having discussed two different retrosynthetic disconnections in the total synthesis of **6**, we will now look at the initially discussed semisynthesis by the Giannis group in 2009 [21] and have a comparison to the Liu/Qin approach from 2024 [26]. This group proposed a divergent semisynthetic approach toward cyclopamine and veratramine, and we will first have a look at their approach toward cyclopamine (see veratramine-type paragraph for the discussion of their reported veratramine synthesis).

**Scheme 11:**
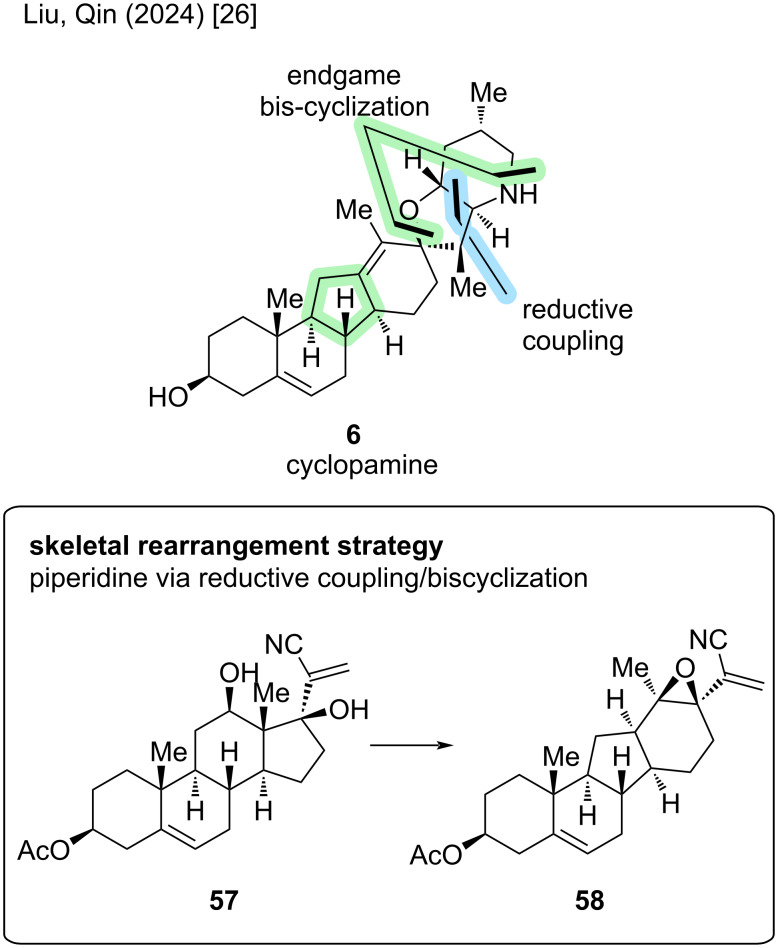
Key steps in the synthesis of cyclopamine (**6**) by the Liu/Qin group [26].

At first glance, the skeletal rearrangement strategy from androstane backbone in **57** to C-*nor*-D-*homo* framework in **58** resembles the key Wagner–Meerwein-type rearrangement by the Giannis group, but also includes significant difference (Scheme 11). First of all, in the Giannis synthesis, the lactone moiety was already installed in the rearrangement step, which was not the case here. The Liu/Qin group decided to perform a reductive coupling after the rearrangement and then close the spiro-E- and piperidine F-rings late-stage. Also, the biomimetic 1,2-alkyl shift serves as the point of divergence in the synthesis of cyclopamine and veratramine, with the hydroxy group at C17 playing a crucial role trapping a cationic intermediate from this rearrangement.

**Scheme 12:**
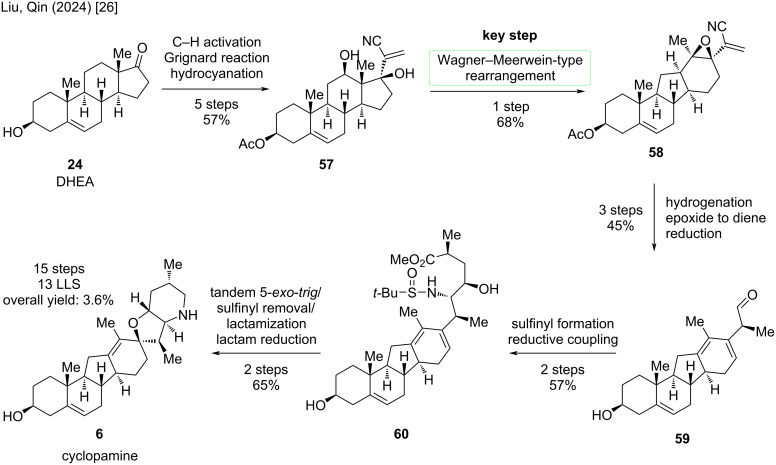
Overview of the semisynthesis of cyclopamine (**6**) by the Liu/Qin group in 2024 [26].

Starting point in this synthesis is again dehydroepiandrosterone (**24**), which was protected in 3-position with an acetyl group (Scheme 12). A copper-mediated C–H activation procedure to obtain the hydroxy moiety in C12 was performed, followed by Grignard addition of ethynylmagnesium bromide and then hydrocyanation to result in vinyl nitrile **57** over five steps. The key Wagner–Meerwein-type rearrangement occurred in 68% yield furnishing epoxide **58**. Hydrogenation of the alkene moiety as well as transformation of the epoxide to a diene in the D-ring (with tetramethyldiamidophosphoric acid chloride) and reduction of the cyano-group gave triene **59** in 45% yield over three steps. The acetyl group was also cleaved in the reduction step. For forging the E- and F-ring of cyclopamine, sulfinyl formation and reductive coupling with an aldehyde made accessible β-amino alcohol **60** over two steps. A cascade reaction occurred upon treatment with HCl in acetone followed by treatment with KOH. Indeed, a tandem 5-*exo*-*trig* cyclization, sulfinyl removal, and lactamization occurred in one-pot. Finally, reduction of the lactam furnished cyclopamine (**6**) in 65% yield over two steps. The reported semisynthesis of the Liu/Qin group displays the shortest route to cyclopamine with 13 steps in the LLS, 15 steps in total and an overall yield of 3.6%.

#### Jervine

**Scheme 13:**
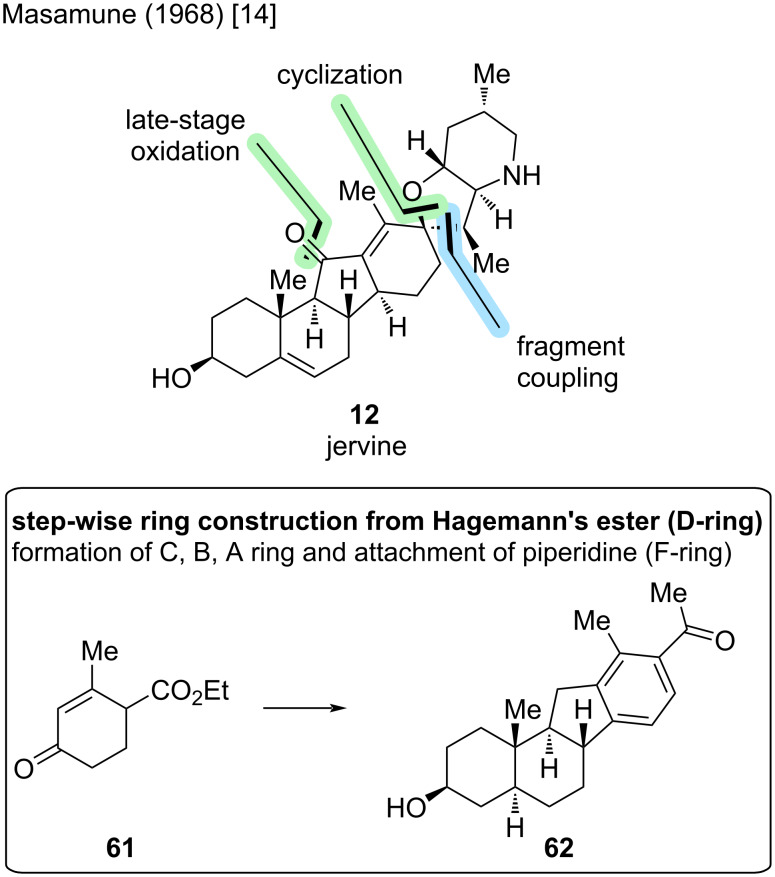
Key steps in the synthesis of jervine (**12**) by the Masamune group [14].

For the C11-oxidized analogue of cyclopamine, jervine (**12**), only one synthesis is reported [14]. The Masamune group disclosed this synthesis in 1968, starting with a sequential ring construction of the C, B and A-ring from Hagemann’s ester (**61**) to C-*nor*-D-*homo* steroid precursor **62** (Scheme 13). The piperidine F-ring was then coupled after D-ring saturation, resulting in a spirocyclization, which we have seen in strategies toward cyclopamine before.

**Scheme 14:**
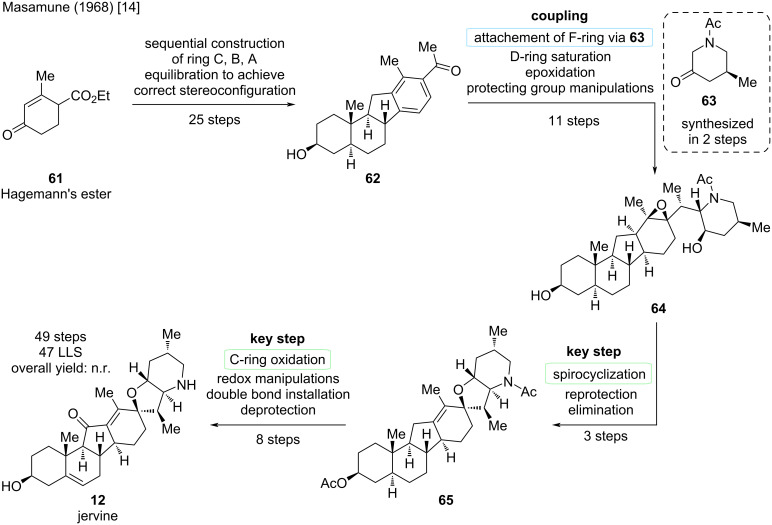
Overview of the total synthesis of jervine (**12**) by the Masamune group in 1968 [14].

As already stated, the synthesis commenced with the sequential construction of the ABCD-ring system in 25 steps to arrive at ketone **62** (Scheme 14). The F-ring was attached via condensation with piperidine **63**. The D-ring was saturated and the remaining alkene epoxidized – a strategy that we have observed in cyclopamine synthesis before. The sequence needed several protecting group manipulations. Therefore, epoxide **64** was synthesized in eleven steps from ketone **62**. The key spirocyclization was performed, which provided the need for reprotection of the secondary amine moiety, and the alcohol was eliminated to furnish **65** in three steps. Compound **65** already closely resembles the desired target **12** but lacks the oxidation at C11 and the double bond in position C5–C6. Eight more steps were needed for the oxidation and the double bond installation mentioned, including several redox manipulations and protecting group removals. In total, the Masamune group reported the total synthesis of jervine (**12**) from Hagemann’s ester (**61**) in 47 steps in the LLS and a total of 49 steps. Not all yields were reported, so no overall yield can be given.

Even though being a much older synthesis, we can create a link to today’s synthesis. The D-ring saturation was also employed in the synthesis by Zhu/Gao [25], with the epoxidation/spirocyclization strategy being closely related, too. In direct comparison, we can observe the effect of today’s methods in comparing to this strategy, while the Masamune group had to choose from much more limited methodology, resulting in tedious protecting group strategies, several reprotections, and redox manipulations [14].

### Comparison of strategies for the jervanine-type

In total, cyclopamine (**6**) has been synthesized four times, two times in a semisynthetic fashion and another two times via total synthesis [21,23,25–26]. Jervine (**12**) was synthesized once [14]. We will only compare the cyclopamine syntheses described. A short comparison of the Masamune synthesis of jervine has been given in the paragraph above, though. Additionally, the comparison of this almost 60-year-old synthesis seemed rather anachronistic and diminished the effort being nothing less than exceptional at that time. Notably, not all yields and conditions were reported, which also made a comparison difficult.

**Scheme 15:**
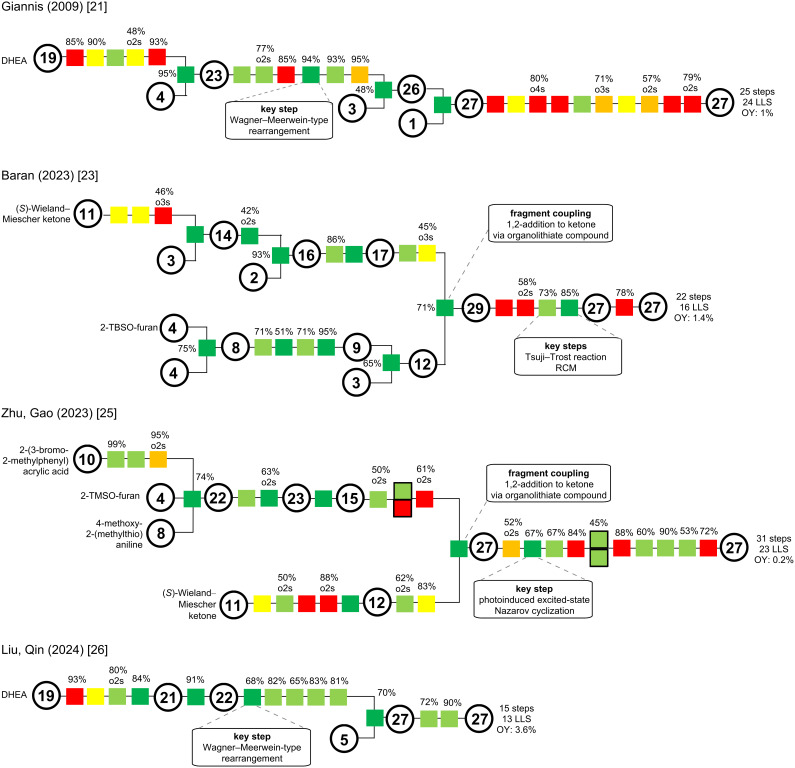
Color-coded schemes of the presented cyclopamine (**6**) syntheses by Giannis, Baran, Zhu/Gao, and Liu/Qin groups [21,23,25–26]. LLS: longest linear sequence; OY: overall yield, RCM: ring closing metathesis.

For an overall comparison of the strategies in the synthesis of jervanine-type natural products, we aimed to achieve a visual comparison via color-coded schemes proposed by the Christmann group (Scheme 15) [10].

In direct comparison, the Giannis synthesis of cyclopamine [21] as the oldest synthesis and as a semisynthesis, poses some non-strategic redox transformations (orange) and protecting manipulations (red) especially in the beginning and in the endgame of the synthesis. Nevertheless, several transformations are desirable green transformations, although it is noticeably more difficult to achieve C–C connections (dark green) in a semisynthesis (with most of the required carbon atoms being already part of the starting material). Highlight of this synthesis was the key biomimetic rearrangement in the middle of the sequence, furnishing the C-*nor*-D-*homo* scaffold. This synthesis provided the first synthetic entry to cyclopamine (**6**) in history and stood unrivaled as a testament for the ingenuity of the Giannis group for more than a decade, which is especially noteworthy with respect to the high biological and medicinal relevance of the target.

This overview allows for a direct comparison to the semisynthesis of Liu/Qin [26] 15 years later. The only non-green transformations displayed at the start were a protecting group installation (red) and a condensation (yellow), which was needed for the C–H activation (light green). All other transformations connect either C–C or C–heteroatom bonds or are strategic redox manipulations. Key step of this semisynthesis was a similar rearrangement, albeit conducted on a diol and leading to an epoxide.

The total synthesis of the Baran group [23] showcases a rather ideal and convergent synthesis. Clearly identifiable are steps in the synthesis, which can further be improved, nevertheless a very high percentage of transformations are highly desirable in achieving complexity. Protecting group manipulations in the endgame could not be entirely avoided and resemble the Giannis group’s strategy. Key in this sequence was the fragment coupling via 1,2-addition of an organolithium species to a ketone fragment, and the key steps with an alkoxide Tsuji–Trost reaction and a ring closing metathesis furnished closing of the D- and spiro-E-ring.

In contrast, the longer synthesis of Zhu/Gao [25] does not display the level of convergence and also poses several undesirable transformations in the synthesis of both fragments, as well as in the later stages of the synthesis. This total synthesis of cyclopamine provided implementation of new methodologies and as a highlight, the key photoinduced excited-state Nazarov cyclization, which installed two stereocenters simultaneously. Notably, both total syntheses commenced from (*S*)-Wieland–Miescher ketone, but made the retrosynthetic cut elsewhere in the molecule (Baran: D-ring, Zhu/Gao: C-ring).

In comparison, a direct evolution can be seen from 2009 to 2024. While the first synthesis of cyclopamine by Giannis [21] stood alone for more than a decade, it still qualifies as the benchmark. Comparing both semisyntheses, one can note the similarities, but also improvements en route with a biomimetic rearrangement effectively reducing the step count in the 2024 semisynthesis by Liu/Quin [26]. Furthermore, two total syntheses successfully tackled cyclopamine as their target, and both elegantly showcase the use of modern synthetic methodology and convergence in retrosynthetic analysis.

### Synthesis in the veratramine subclass

In the veratramine-type class of *Veratrum* alkaloids, two structures have been revisited for total synthesis approaches: veratramine (**13**) and verarine (**14**). In comparison, verarine lacks the hydroxy moiety at C23 in the F-ring (see Scheme 4).

#### Veratramine

In the 1950s case, another *Veratrum* alkaloid was found in *Veratrum californicum* and responsible for leg-deformations that were observed in the newborn lambs, in addition to the already discussed single-eyed phenotype: veratramine (**13**) [7]. Veratramine was determined to be the degradation product of acid-sensitive cyclopamine (**6**) [35].

**Scheme 16:**
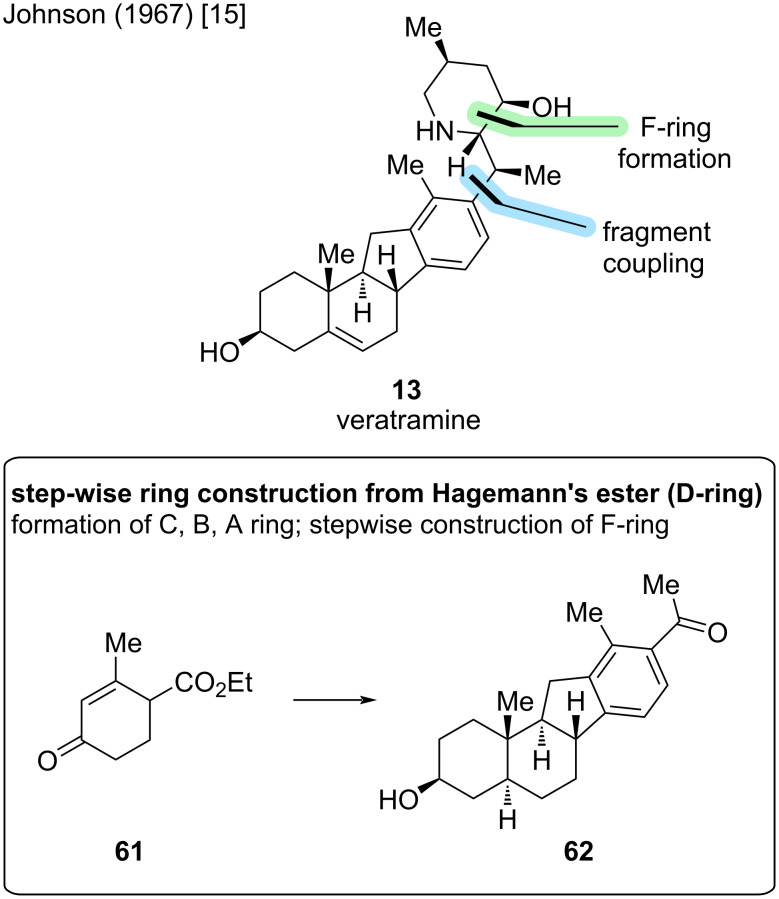
Key steps in the total synthesis of veratramine (**13**) by the Johnson group [15].

The first reported synthesis of veratramine (**13**) was published by the Johnson group in 1967 [15]. This synthesis starts from Hagemann’s ester (**61**) and sequentially constructs the ABCD-steroidal skeleton. This synthesis was already presented in the total synthesis of jervine [14]. More precisely, the Masamune group adopted this sequence from **61** to **62** in 1968 from the synthesis of Johnson described below. Introduction of the F-ring was performed by attachment of an alkyl chain, which was later cyclized to the desired piperidine ring (Scheme 16).

**Scheme 17:**
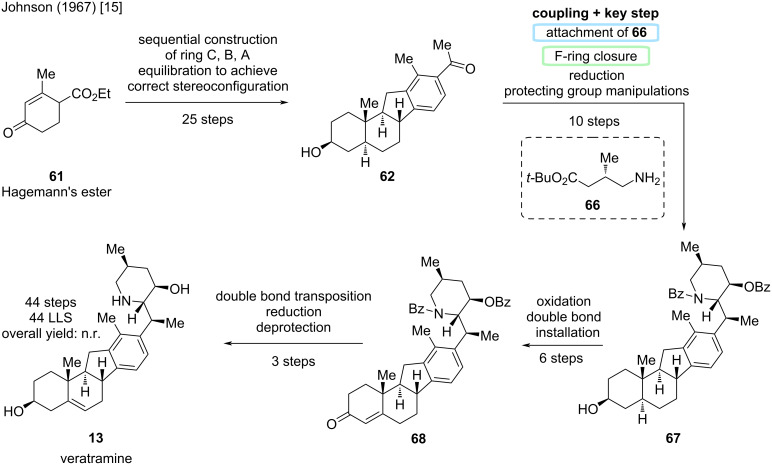
Overview of the total synthesis of veratramine (**13**) by the Johnson group in 1967 [15].

25 steps were needed for the construction of the ABCD-ring system in **62** (Scheme 17). Attachment of amine **66**, F-ring closure, a reduction and protecting group manipulations led to structure **67** in 10 steps, which already represented the core veratramine skeleton, but lacked the C5–C6 double bond. For this, six more steps were needed to oxidize the C3-alcohol and install a C4–C5 double bond to arrive at enone **68**. In three steps, the double bond was transposed to the correct position, the ketone reduced, and the remaining protecting groups removed. The Johnson group, thus achieved the first total synthesis of veratramine (**13**) in 44 steps (LLS), which is also the total step count in completely linear sequence.

We will now revisit two already discussed total syntheses: the Zhu/Gao synthesis of 2023 [25], which proceeds through veratramine (**13**) to access cyclopamine (**6**) and the semisynthesis of Liu/Qin of 2024 [26], which provides divergent access to cyclopamine (**6**) and veratramine (**13**). Additionally, the Trauner group published another total synthesis of **13** in 2025 [27].

**Scheme 18:**
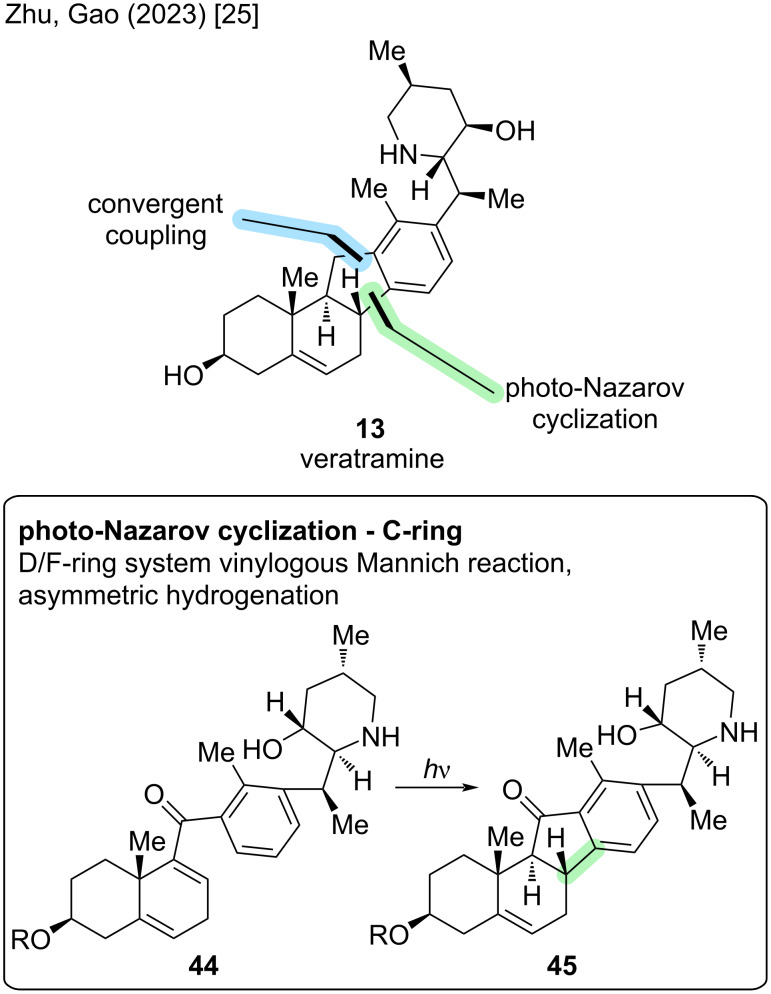
Key steps in the synthesis of veratramine (**13**) by the Zhu/Gao group [25].

The major retrosynthetic cut in the Zhu/Gao group synthesis of veratramine [25] proceeds through the C-ring (Scheme 18). Key highlight of this synthesis poses the photoinduced excited-state Nazarov cyclization (**44** → **45**). The cut for the convergent coupling divides the molecule in a left-hand AB-fragment with an aldehyde, while the right-hand fragment is an aryl bromide (D/F ring structure).

**Scheme 19:**
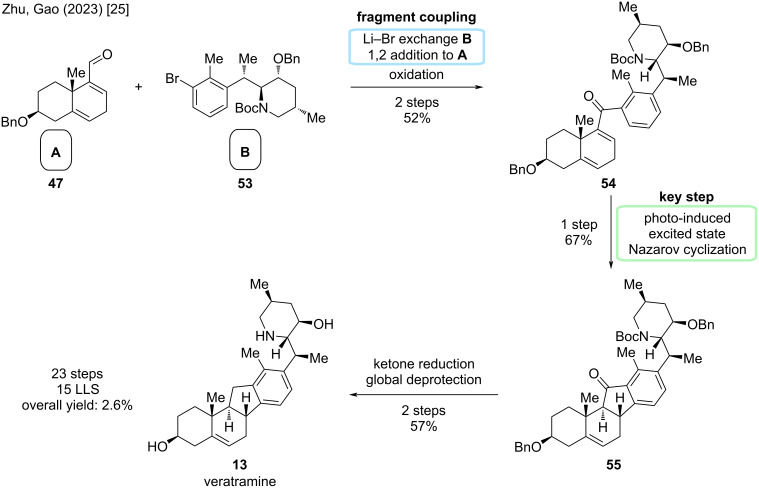
Shortened overview of the total synthesis of veratramine (**13**) by the Zhu/Gao group in 2023 [25].

Fragment A was synthesized from (*S*)-Wieland–Miescher ketone (**34**) via an eight-step sequence (Scheme 19). Fragment B was synthesized from a substituted acrylic acid in 10 steps toward aryl bromide **53**. Key success factor in this sequence was an asymmetric hydrogenation developed by the same group and an enantioselective vinylogous Mannich reaction. For a more detailed explanation of the synthesis of these fragments, the reader is referred to the synthesis of cyclopamine (**6**) in the previous paragraph (Scheme 10).

Convergent coupling was carried out via lithium–halogen exchange, 1,2-addition, and subsequent oxidation to give ketone **48**. Photoinduced excited-state Nazarov cyclization installed the cyclopentenone C-ring with correct stereoconfiguration at C9 and C10 in excellent yield. Ketone reduction and global deprotection gave veratramine (**13**) in two more steps. In conclusion, this provided an entrance to veratramine in 15 steps LLS (23 steps total, overall yield 2.6%), while also providing cyclopamine in a relay synthesis in eight more steps.

**Scheme 20:**
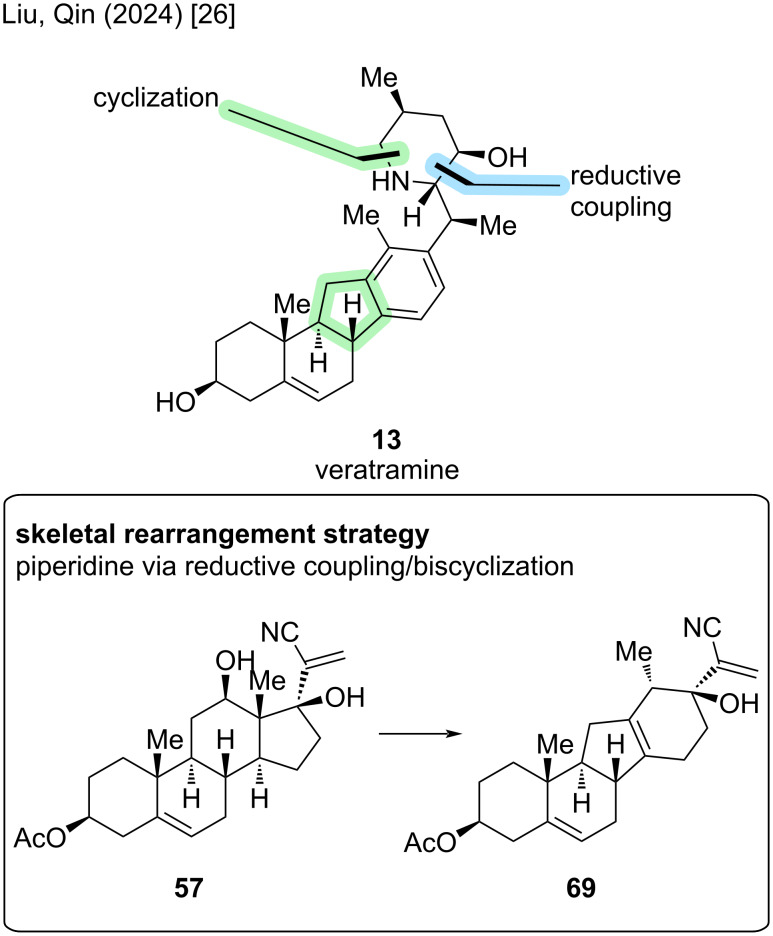
Key steps in the synthesis of veratramine by the Liu/Qin group [26].

Another synthesis of cyclopamine will be revisited: We discussed the divergent semisynthesis of Liu/Qin toward cyclopamine, but until this point, left out the synthesis of veratramine [26]. The key steps rely on a Wagner–Meerwein-type rearrangement; the deviation occurs after this step aiming at the aromatization of the D-ring (Scheme 20).

**Scheme 21:**
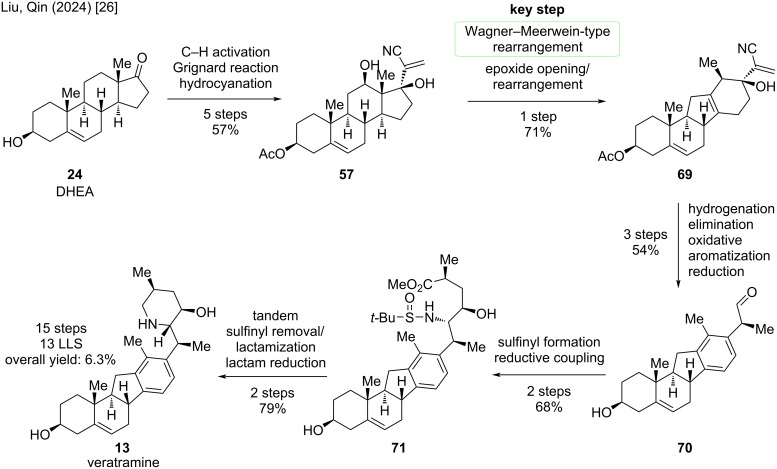
Overview of the semisynthesis of veratramine (**13**) by the Liu/Qin group in 2024 [26].

Starting from dehydroepiandrosterone (**24**), DHEA, previously discussed five steps were performed to give diol **57** (Scheme 21). In the synthesis of cyclopamine, the Wagner–Meerwein-type rearrangement yielded an epoxide at C13–C17 in the presence of Tf_2_O and 2-chloropyridine at −78 °C. By treatment of this epoxide with methanesulfonic acid, presumably through an epoxide opening, 1,2-hydride shift, and deprotonation, alcohol **69** was obtained. These two transformations were combined to achieve rearrangement of **57** to **69** in 71% in one single step. The *exo*-methylene group was selectively hydrogenated, the C17-alcohol eliminated, and then the D-ring oxidatively aromatized by the action of ceric ammonium nitrate. Reduction of the nitrile moiety furnished aldehyde **70**, which was obtained on gram-scale over three steps in 54%. The endgame of this synthesis closely resembles the one of cyclopamine (Scheme 12). Sulfinyl formation and reductive coupling lead to β-amino alcohol **71**, which was then transformed through similar conditions (see cyclopamine paragraph) via a tandem sulfinyl removal/lactamization, and subsequent lactam reduction, to veratramine (**13**). In particular, this semisynthesis furnishes veratramine in 13 steps in the longest linear sequence, a total step count of 15 steps, and an overall yield of 6.3%. To date, this is the shortest and highest-yielding synthesis of veratramine from a commercially available starting materials.

**Scheme 22:**
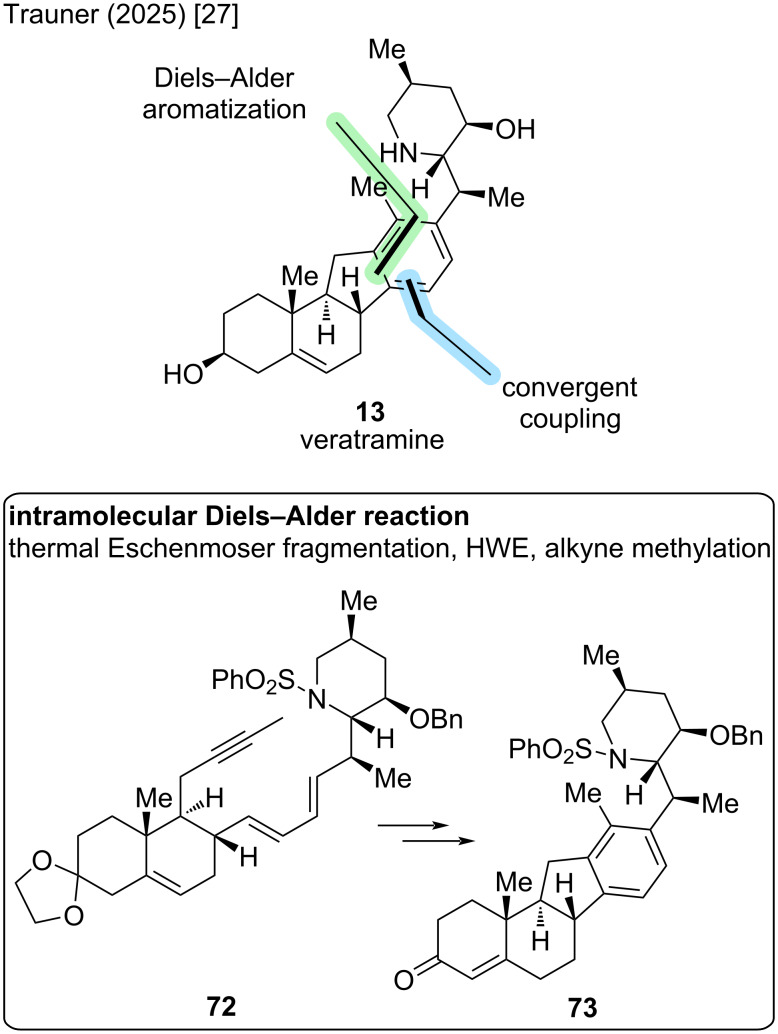
Key steps in the synthesis of veratramine (**13**) by the Trauner group [27].

Another synthesis that comes very close to the previous results is the recently published synthesis by the Trauner group [27]. In comparison to the Liu/Qin group [26], it provides a total synthetic entry in 13 steps in the longest linear sequence and 19 steps in total. The key disconnection cuts across the D-ring through a Diels–Alder reaction and aromatization (Scheme 22). The coupling occurs in C14–C15 via a Horner–Wadsworth–Emmons (HWE) reaction. Interestingly, the left-hand fragment is synthesized in an unusual thermal Eschenmoser fragmentation, the product being directly conjoined in a one-pot fashion.

**Scheme 23:**
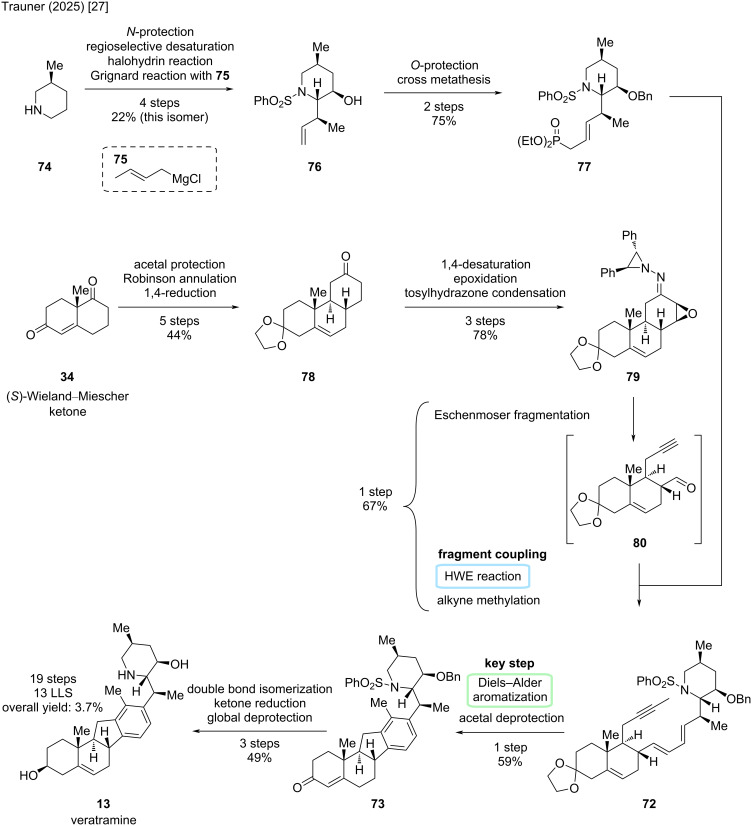
Overview of the total synthesis of veratramine (**13**) by the Trauner group in 2025 [27].

First, commercially available (*S*)-3-methylpiperidine (**74**) was *N*-protected and regioselectively desaturated to furnish known phenylsulfonylenamine (Scheme 23). Conversion toward the bromohydrin, treatment with potassium hydride, and subsequent addition of the crotyl-Grignard reagent **75** gave the desired isomer **76** in 22% yield over four steps. More exactly, the diastereomeric piperidine was obtained in a 1:1 ratio, and the Trauner group also finished the synthesis of 20-*iso*-veratramine with this diastereomer, which is not included herein. Protection of the hydroxy moiety and cross metathesis furnished the right-hand fragment **77** in two more steps.

For the construction of the ABC-skeleton in ketone **78**, the (*S*)-Wieland–Miescher ketone (**34**) was subjected to acetal protection, a Robinson annulation, and 1,4-reduction in five steps and 44% yield. In the next three steps, a 1,4-desaturation gave a net double bond transposition to C13–C14, followed by an epoxidation and tosylhydrazone formation to furnish **79** in excellent yield. The key connection in this synthesis proceeded in a one-pot fashion in 67% yield. At first, tosylhydrazone **79** was subjected to a thermal Eschenmoser fragmentation at 150 °C under microwave conditions to obtain both, an alkyne and aldehyde moiety in intermediate **80**. The crude material was concentrated and subjected to a Horner–Wadsworth–Emmons (HWE) reaction with phosphonate **77**. In the same pot, the alkyne moiety was also methylated to furnish dienyne **72**. Now, the key Diels–Alder step could be performed. Initial attempts failed, but a rhodium-catalyzed reaction in trifluoroethanol enabled the desired [4 + 2]-addition. Some aromatization as well as ketal deprotection was observed, so by optimization, AgSbF_6_ and diethyl fumarate enhanced this reactivity, while this modification also significantly improved the yield to 59% of this step. It was noted that the silver salt helped to abstract the chloride of the [Rh(cod)Cl]_2_ catalyst, forming a cationic rhodium complex in situ, while diethyl fumarate acted as a hydrogen acceptor. Double bond isomerization, ketone reduction and deprotection of **73** furnished veratramine (**13**). This Diels–Alder strategy concluded the synthesis of veratramine in 13 steps LLS (19 steps total, overall yield of 3.7%).

Similar Diels–Alder strategies to construct the D-ring, e.g., a Diels–Alder reaction in the synthesis of 4-methylenegermine (Stork, 2017) [22] and an intramolecular Diels–Alder reaction with furan for several congeners (Luo, 2023 and 2024) [29–30], can later be found in the cevanine-type paragraph.

#### Verarine

**Scheme 24:**
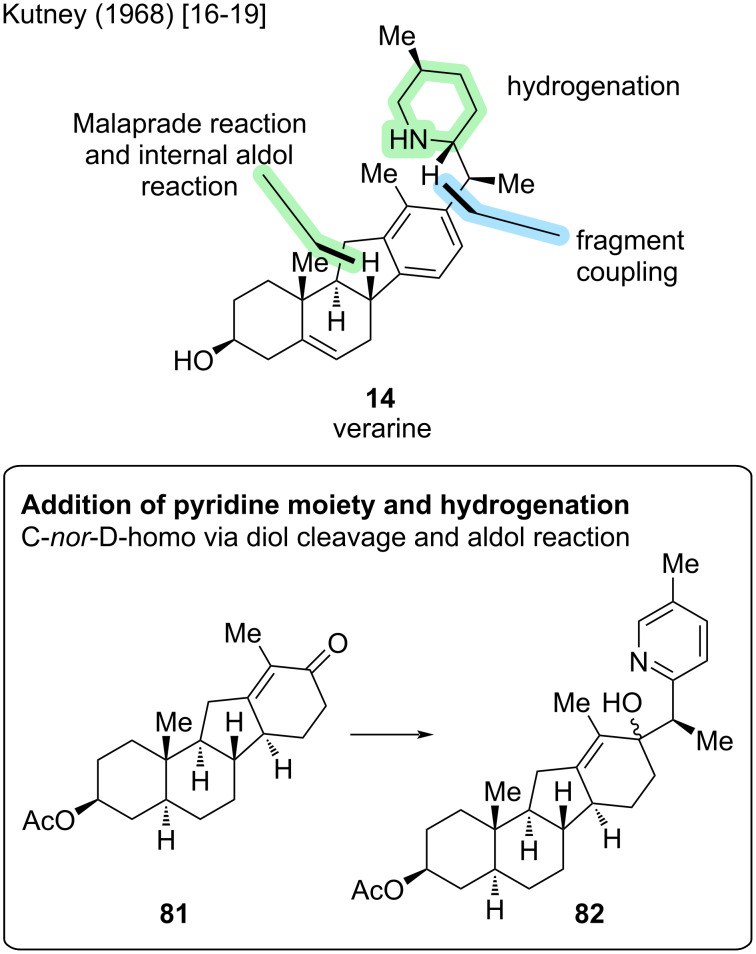
Key steps in the synthesis of verarine (**14**) by the Kutney group [16–19].

The synthesis of the deoxygenated congener of veratramine, verarine (**14**), was accomplished in 1968 by the Kutney group [19]. This group published the sequential synthesis in four separate papers in 1962, 1963, 1965, and 1968 [16–19]. The earlier publications provide an access to the ABCD-skeleton with a C-*nor*-D-*homo* motif through a diol cleavage (Malaprade reaction) and intramolecular aldol reaction to furnish the five-membered C-ring. The F-ring is later attached, first as a pyridine, which then gets hydrogenated to a piperidine (Scheme 24).

**Scheme 25:**
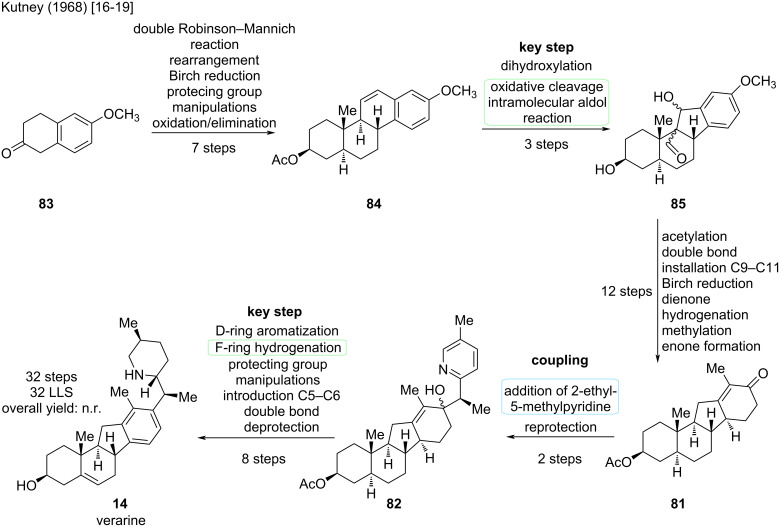
Overview of the total synthesis of verarine (**14**) by the Kutney group reported 1962–1968 [16–19].

This sequence starts from 6-methoxy-2-tetralone (**83**), which was transformed in seven steps into intermediate **84** (Scheme 25). Important was the double bond at C13–C14, which was then dihydroxylated, the so-obtained diol was cleaved, and an intramolecular aldol reaction furnished the rearranged scaffold **85** in three steps. Acetyl protection and elimination at C9–C11, followed by a Birch reduction, yielded a dienone structure. The latter was then hydrogenated, a methyl group was installed in α-position to the ketone, and an enone was formed to give **81** over 12 steps. Then, 2-ethyl-5-methylpyridine was added to the intermediate to yield **82**. Finally, the D-ring was aromatized, the pyridine ring hydrogenated, and after several protecting group manipulations, installation of the C5–C6 double bond and deprotection, verarine (**14**) was obtained. The reported synthesis of verarine has a total step count of 32 steps, displaying an entirely linear route.

### Comparison of strategies for the veratramine-type

**Scheme 26:**
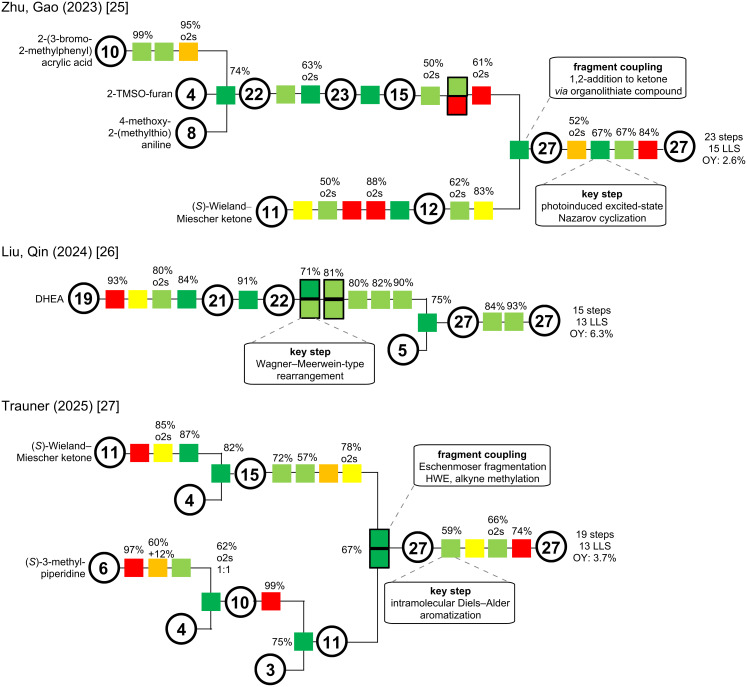
Color-coded schemes of the presented veratramine-type alkaloid synthesis of Zhu/Gao, Liu/Qin and Trauner groups [25–27]. LLS: longest linear sequence; OY: overall yield.

In summary, veratramine (**13**) has been synthesized four times, first in 1967, by the Johnson group [15], two times by total synthesis [25,27], and once in a semisynthetic fashion [26]. A synthesis of the closely related verarine (**14**) was reported in 1968 by the Kutney group [19]. For our comparison using the color-coded Christmann scheme [10], we will stick to our proposal and only display the syntheses from this millennium, while also acknowledging the efforts of the Johnson group (1967) [15] and the Kutney group (1961–1968) [16–19] in their respective era. Scheme 26 displays the color-coding for the syntheses of veratramine (**13**) reported by the Zhu/Gao [25], the Liu/Qin [26] and the Trauner groups [27].

In direct comparison, the Liu/Qin semisynthesis [26] of veratramine stands out, displaying an almost linear sequence. Featuring a key Wagner–Meerwein-type rearrangement from a diol to an homoallylic alcohol, most transformations are on the green and, thus, desirable spectrum of our color code. Note that a semisynthetic approach always lacks real C–C bond connection (dark green). In particular, this synthesis provides a fast and gram-scale access to veratramine.

The total synthesis by the Zhu/Gao group [25] has a high-level of convergence, but includes protecting group manipulations (red). Key step of this sequence was the photo-Nazarov cyclization close to the end of the synthesis.

The Trauner group [27] published an even more convergent synthesis, rapidly assembling the complex structure of veratramine in a very short sequence (13 steps LLS, compare to 13 steps in Liu/Qin semisynthesis). The fragment coupling was performed through a thermal Eschenmoser fragmentation, a Horner–Wadsworth–Emmons (HWE) reaction and an alkyne methylation, all proceeding in one-pot. This then set the stage for the key Diels–Alder/aromatization sequence.

Although three entirely different approaches are displayed, all three pose a fast 13 or 15 step longest linear sequence access to veratramine. Shortly published one after another, we can witness a disconnection through the C-ring (Zhu/Gao) [25], a rearrangement strategy (Liu/Qin) [26] and a cross-D-ring disconnection (Trauner) [27], leading to a short and concise synthesis with state-of-the-art methodology.

### Synthesis in the cevanine subclass

The cevanine-type alkaloids constitute the largest of the three subgroups of *Veratrum* alkaloids. However, they have long withstood the efforts of total synthesis endeavors, perhaps owing to their typically densely functionalized hexacyclic framework. To date, the isolation of at least 80 different cevanine-type alkaloids has been reported [13,38–42]. The common hexacyclic framework is often highly oxidized and the hydroxy groups are further functionalized as esters of various carboxylic acids. Apart from the hydroxylation pattern, structural diversity is also imparted through a *cis*- or *trans*-fusion of the A,B-rings and the configuration of the methyl group at C25, among other features [13].

**Scheme 27:**
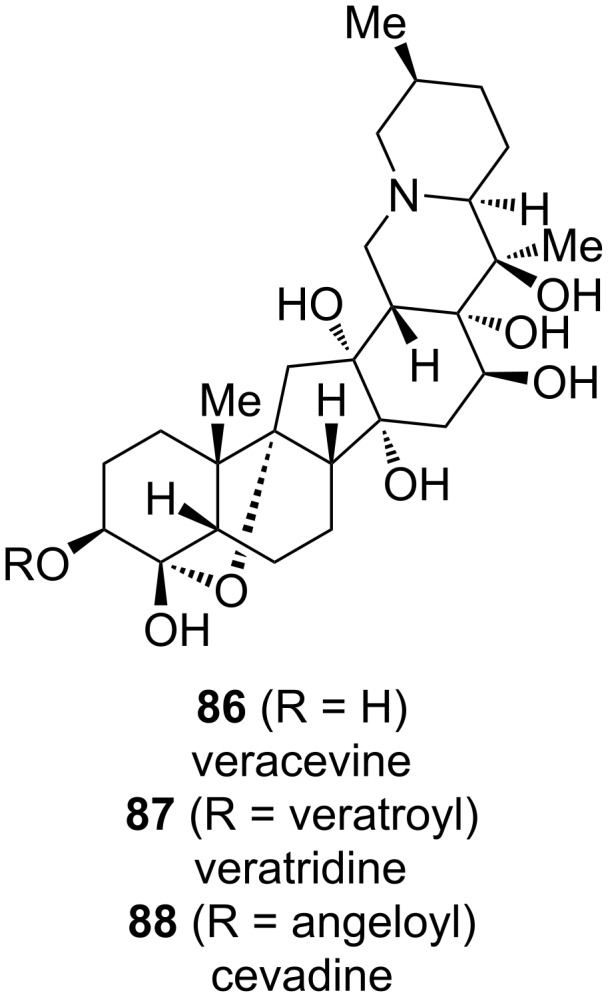
Structures of veracevine (**86**), veratridine (**87**), and cevadine (**88**).

Like the related subclasses, cevanine-type alkaloids exhibit toxicity in humans by acting on the sodium channels of nerve cells. Increased stimulation, associated with the vagal nerve results in a reflex that causes the triad of responses known as the Bezold–Jarisch reflex: hypotension, bradycardia, and hypopnea [43]. It is proposed that the death of Alexander the Great (June 11th, 323 BC) was caused by *Veratrum* poisoning, more specifically, extracts from *Veratrum album*. This hypothesis is based on the symptoms and timeframe described in the Alexander Romance, which is only one of two divergent accounts describing Alexander’s death [44]. Veratrine, a highly toxic plant extract, contains a mixture of alkaloids with the two major constituents being veratridine (**87**) and cevadine (**88**), both cevanine-type alkaloids derived from the core alkamine veracevine (**86**) (see Scheme 27) [45]. Extracts containing *Veratrum* alkaloids have been explored as potential treatments for hypertension. However, their narrow therapeutic window led to their replacement by other medications [43].

#### Verticine

The first cevanine-type *Veratrum* alkaloid to be successfully synthesized in the laboratory is (−)-verticine (**15**) (sometimes also called peimine). In 1977, Kutney and co-workers reported its semisynthesis from hecogenin acetate (**91**) in 30 steps [20,46]. It was only until four decades later that chemical methods developed to a point rendering the de novo total synthesis of cevanine-type *Veratrum* alkaloids feasible.

**Scheme 28:**
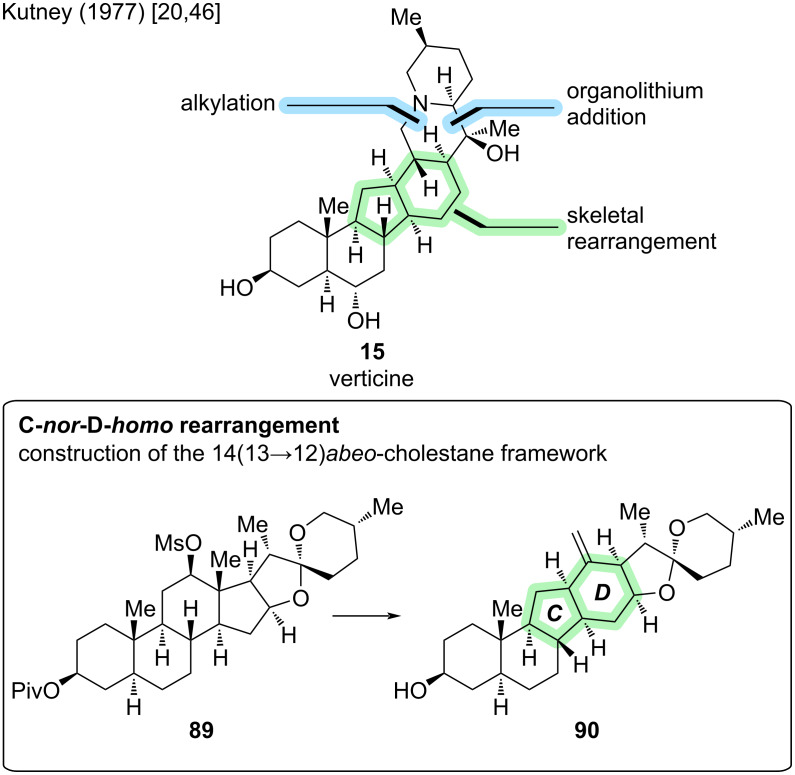
Key step in the semisynthesis of verticine (**15**) by the Kutney group (1977) [20,46].

In accordance with the biosynthetic pathway [47], the C-*nor*-D-*homo* skeleton of **15** was constructed using a cationic rearrangement of a steroid precursor (see Scheme 28).

**Scheme 29:**
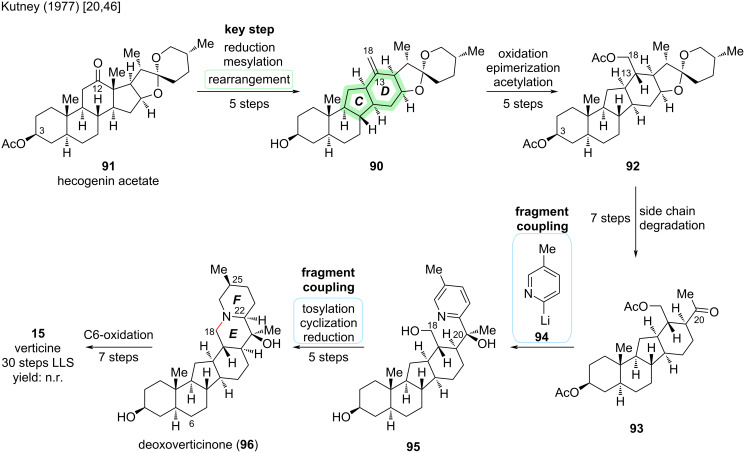
Overview of the semisynthesis of verticine (**15**) by the Kutney group (1977) [20,46].

Hecogenin acetate (**91**) was first transformed into rockogenin 12-methanesulfonate-3-pivalate (**89**) by reduction and mesylation. Heating to reflux in anhydrous pyridine afforded the rearrangement to the C-*nor*-D-*homo* isosteroid skeleton **90** with an exocyclic 13,18-double bond in 5 steps from hecogenin acetate (see Scheme 29). In a five-step sequence of redox manipulations and acetylation, olefin **90** was transformed into 3,18-diacetate **92**. The degradation of the superfluous side chain was realized by acidic cleavage of the spiroketal moiety, followed by redox manipulations. A seven-step sequence afforded methyl ketone **93** [20].

The F-ring was introduced by addition of 2-lithio-5-methylpyridine (**94**) to methyl ketone **93**, with the major addition product **95** exhibiting the desired configuration at C20. Tosylation of the primary C18–OH in **95** (and secondary C3–OH) was followed by intramolecular nucleophilic substitution through the pyridine nitrogen. The pyridinium species was reduced to reveal the piperidine E-ring with correct configuration of the stereocenters at C22 and C25. Reduction of the C3-tosylate afforded deoxoverticinone (**96**), a known congener of verticine. Seven more redox manipulation steps were required to install the C6–OH group and conclude the semisynthesis of (−)-verticine (**15**) in 30 steps from hecogenin acetate (**91**) [46]. It is noted that for most of the individual steps, no yield was reported, so no overall yield can be calculated. To date, no other synthetic endeavor toward verticine has been disclosed.

#### 4-Methylenegermine

**Scheme 30:**
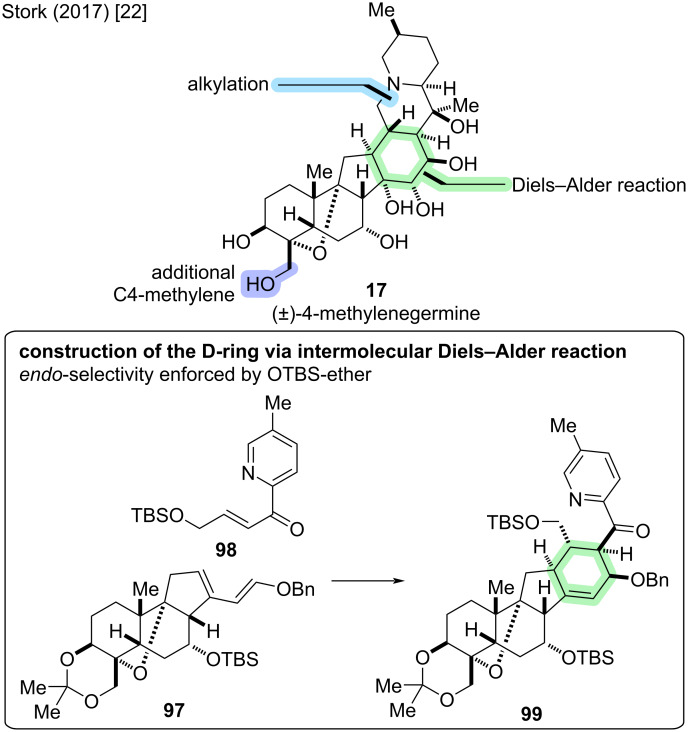
Key step of the total synthesis of (±)-4-methylenegermine (**17**) by the Stork group (2017) [22].

The total synthesis of (±)-4-methylenegermine (**17**) was reported by Stork and co-workers in 2017 [22]. It represents an unnatural derivative of germine (**20**) with an additional methylene unit at C4 that was introduced to increase the stability of synthetic intermediates by avoiding the base-sensitive hemiketal motif. The synthetic plan was to carve out the superfluous hydroxymethyl group in the late stage of the synthesis using a stereoretentive decarboxylative hydroxylation procedure [48]. A key step in the synthesis was an intermolecular Diels–Alder reaction between diene **97** and dienophile **98** to construct the six-membered D-ring (see Scheme 30).

**Scheme 31:**
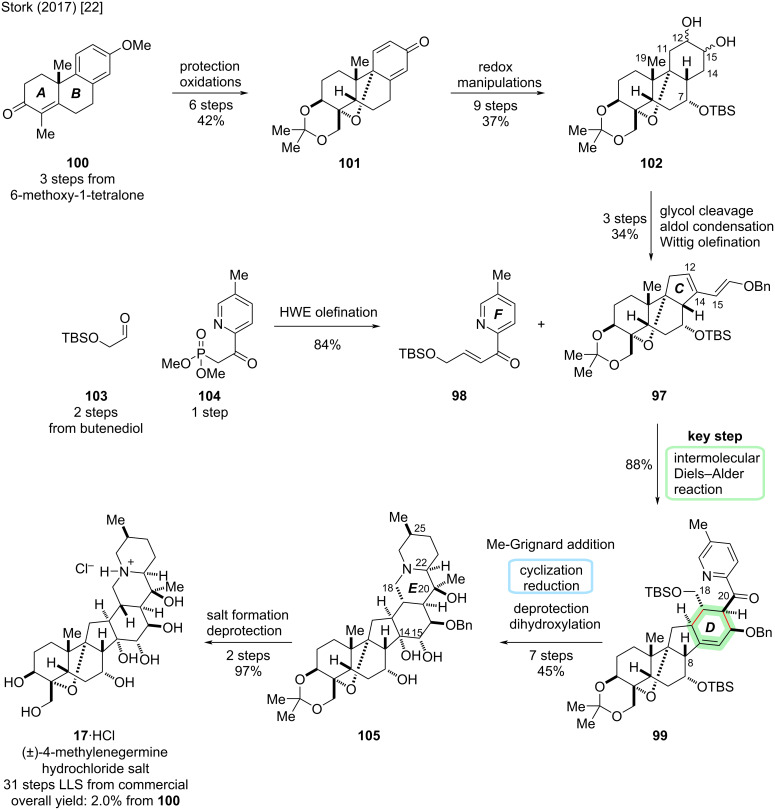
Overview of the total synthesis of (±)-4-methylenegermine (**17**) by the Stork group (2017) [22].

Starting from (racemic) tricyclic ketone **100** (3 steps from commercially available 6-methoxy-1-tetralone) [49], a six-step sequence of oxidative transformations and protecting group manipulations was employed to arrive at dienone **101**, which had the C4/C9 oxo bridge installed (see Scheme 31). Notably, the asymmetric preparation of **100** has also been described (9 steps LLS from commercial substances) [50–51]. The introduction of C7–OH and reduction of double bonds was achieved following a nine-step sequence of redox manipulations to arrive at C12,15-glycol **102**. The glycol was subjected to periodate cleavage, which was followed by intramolecular aldol reaction to effect contraction of the C-ring. The observed regioselectivity was rationalized by steric influence of the C19-methyl group hindering deprotonation at C11. Thus, desired cyclization corresponding to deprotonation at C14 was observed. The elimination to the enal did not occur spontaneously but required heating of the corresponding C12-mesylate, hinting at the ring strain accompanied by formation of the double bond in the *trans*-hydrindane system. The enal was subjected to a Wittig olefination to construct diene **97** for the Diels–Alder reaction.

Dienophile **98** was constructed using a Horner–Wadsworth–Emmons (HWE) reaction between aldehyde **103** (2 steps from butenediol) and phosphonate **104** (1 step from commercially available materials). The intermolecular Diels–Alder reaction was achieved in refluxing toluene, giving **99** in 88% yield as a single diastereomer. Due to steric hindrance, dienophile **98** would approach the more accessible face of diene **97**, i.e., *cis* to C8–H. Furthermore, exclusive *endo*-addition was observed because *exo*-addition would lead to severe interference between the TBSO-substituent on the dienophile and the diene. As a result of such *endo-*addition, the benzyloxy group in the diene and the acylpyridine of the dienophile are oriented *cis* to each other in adduct **99**.

Next up was the formation of the C20–OH by addition of MeMgBr to the ketone **99**, which initially gave the undesired α-alcohol. However, the stereoselectivity of the Grignard reaction could be reversed by previous complexation with the trimethylaluminum salt of BHT to give predominantly the desired β-alcohol. Protecting group manipulations at C20–OH and C18–OH were followed by treatment with TfOH to afford cyclization of the E-ring. The obtained pyridinium species was directly reduced to reveal the piperidine moiety. The observed selectivity for hydrogenation from the α-side was attributed to the steric hindrance of the C20 axial tertiary TMS ether. The survival of the olefin in the D-ring was attributed to the hindrance by the axial benzyl ether group on one side, and the C7 axial TBS ether group on the other. Removal of the silyl groups at C7 and C20 with CsF was followed by dihydroxylation of the C14,15-double bond, and the major product **105** was determined to be the desired α-diol by ^1^H–^1^H-NOESY and DFT calculations. The tertiary amine was masked as the hydrochloride salt with concomitant ketal deprotection, which was followed by hydrogenation to remove the benzyl protecting group to obtain (±)-4-methylenegermine hydrochloride (**17**·HCl) in 31 steps (LLS from commercial materials) and an overall yield of 2.0% from **100** [22].

A plan for the conversion of **105** to (±)-(**20**) was to follow Barton’s conditions for decarboxylative hydroxylation [48]. However, due to lack of material at the late stage, these steps could not be realized. The total synthesis of **20** was later disclosed by Luo and co-workers in a divergent approach to several congeners (vide infra) [30].

#### Heilonine

Heilonine (**16**) represents a rare example of a cevanine-type alkaloid with an aromatized D-ring. Furthermore, the configuration of the stereocenter at C25 is opposite to other previously synthesized members of the group. The isolation of heilonine was reported in 1989 by Kaneko et al. from *Fritillaria ussuriensis*. It is believed to be a constituent of “beimu", a traditional Chinese medicine that is used as a sedative, antitussive, and expectorant [52]. The nine stereocenters reside in two different regions of the molecule separated by the aromatic D-ring. To date, two total syntheses of (+)-**16** have been reported [24,28].

**Scheme 32:**
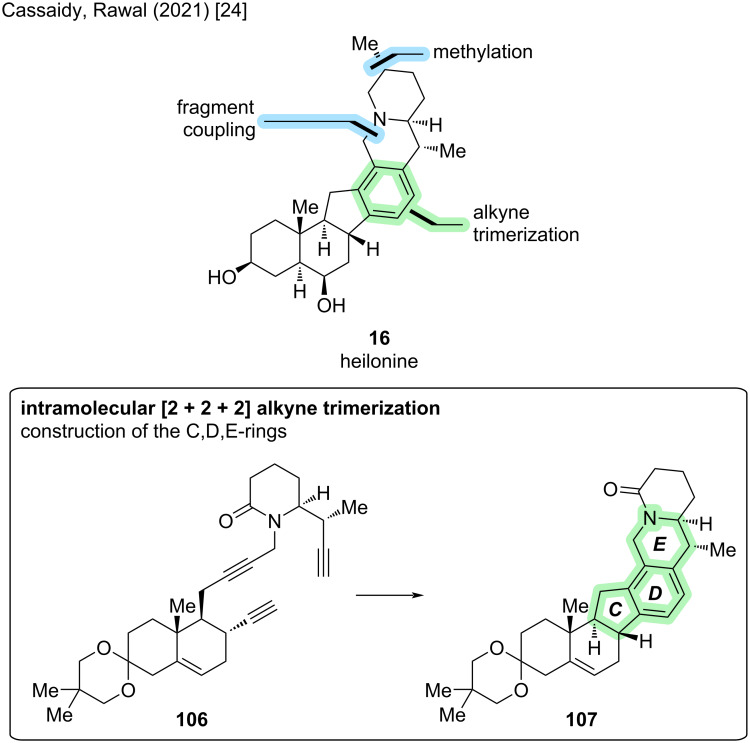
Key step of the total synthesis of heilonine (**16**) by Cassaidy and Rawal (2021) [24].

**Scheme 33:**
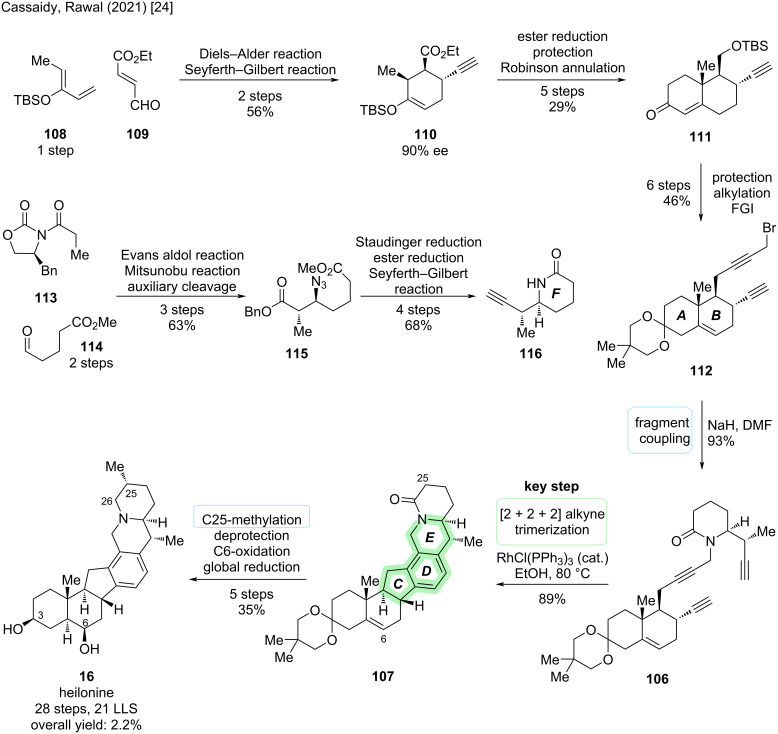
Overview of the total synthesis of heilonine (**16**) by Cassaidy and Rawal (2021) [24]. FGI: functional group interconversions.

The first total synthesis of (+)-**16** was reported in 2021 by Cassaidy and Rawal [24]. At the time, this also represented the first de novo synthesis of a cevanine-type alkaloid (the synthesis by Stork described above “only” afforded the unnatural 4-methylenegermine) [22]. The key step of this synthesis was the construction of the aromatic D-ring via [2 + 2 + 2] alkyne trimerization (see Scheme 32). The corresponding triyne precursor **106** was constructed by a convergent coupling of diyne fragment **112** and alkyne fragment **116** (see Scheme 33).

The synthesis of diyne fragment **112** commenced with an organocatalytic, enantioselective Diels–Alder reaction between siloxydiene **108** (synthesized in one step from ethyl vinyl ketone) and commercially available dienophile **109** using the proline-derived Hayashi ligand [53]. This delivered the desired *exo*-adduct with good ee on a decagram-scale and installed three stereocenters with the correct configuration. The aldehyde moiety was converted to the terminal alkyne **110** via Seyferth–Gilbert homologation using the Ohira–Bestmann reagent. Reduction of the ethyl ester moiety and protection of the primary alcohol was followed by a Robinson annulation with methyl vinyl ketone to construct bicyclic fragment **111**. The enone moiety was protected as an acetal, followed by alkylation of the primary alcohol with a propargyllithium species. Further functional group interconversions were employed to install the propargyl bromide moiety and conclude the synthesis of diyne fragment **112** containing the A,B-rings.

The preparation of alkyne fragment **116** started with an Evans *syn*-aldol reaction between propionic acid Evans auxiliary **113** and aldehyde **114** (prepared in two steps from δ-valerolactone). This was followed by an azide-Mitsunobu reaction and auxiliary removal with LiOBn to yield β-azido ester **115**. Staudinger reduction of the azide moiety with PPh_3_ was accompanied by spontaneous cyclization to the lactam. The benzyl ester was converted to the aldehyde (2 steps), followed by Seyferth–Gilbert homologation with the Ohira–Bestmann reagent to conclude the synthesis of piperidine fragment **116** constituting the later F-ring.

The two chiral fragments were conjoined by alkylation of piperidinone **116** with propargyl bromide **112** to provide triyne **106** in excellent yield. After some optimization studies, RhCl(PPh_3_)_3_ (Wilkinson’s catalyst) in refluxing EtOH was found to be an efficient catalyst to promote the [2 + 2 + 2] cyclotrimerization in high yields in a single step. This transformation constructed not only the aromatic D-ring, but also the C- and E-rings found in heilonine (**16**). Incorporation of the methyl group at C25 with desired configuration was, after some optimization, afforded by alkylation with MeI and LiTMP/HMPA. Four more steps were required for deprotection of the ketal and installation of the correct oxidation levels at C3, C6, and C26 to conclude the first total synthesis of (+)-**16** (28 steps, 21 LLS, 2.2% total yield). The identity of the natural product was confirmed by acetylation and comparison with heilonine diacetate [24].

**Scheme 34:**
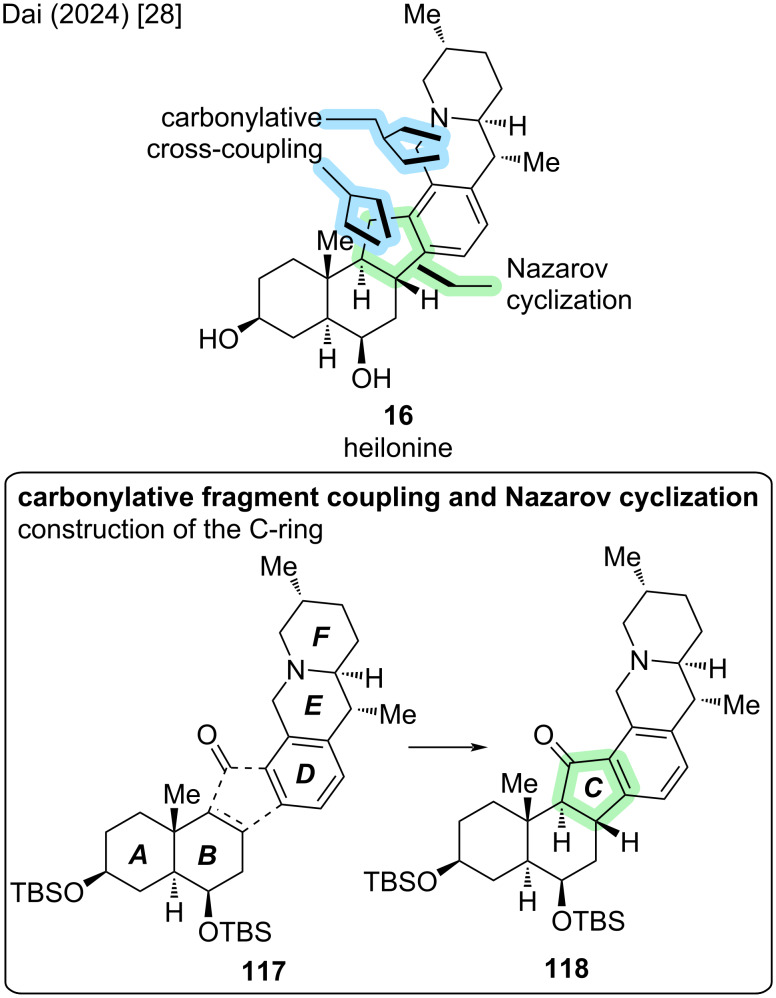
Key steps of the synthesis of heilonine (**16**) by Dai and co-workers (2024) [28].

**Scheme 35:**
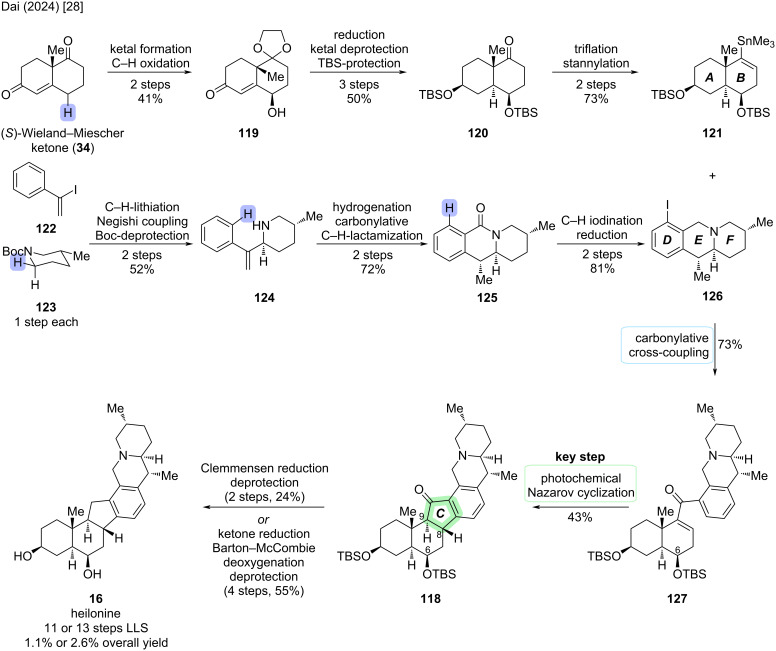
Overview of the total synthesis of heilonine (**16**) by Dai and co-workers (2024) [28].

Three years later, Dai and co-workers described the second enantioselective total synthesis of (+)-**16** by harnessing the power of C–H-functionalization transformations [28]. A key disconnection in their approach was a Nazarov cyclization to construct the five-membered C-ring (see Scheme 34). The corresponding dienone precursor **127** was synthesized by a convergent, carbonylative cross-coupling of two fragments, the AB-fragment **121** and tricyclic DEF-fragment **126** (see Scheme 35).

The synthesis of the AB-fragment **121** commenced with the ketal protection of (*S*)-Wieland–Miescher ketone (**34**). This was followed by Na_2_-eosin Y-catalyzed visible-light-induced γ-hydroxylation to furnish γ-hydroxyenone **119**. Reduction and protecting group manipulations afforded bis-TBS-ether **120**, which was converted to vinylstannane **121** in two steps via the corresponding vinyl triflate.

The synthesis of the DEF-fragment **126** commenced with a Boc-directed C–H-lithiation of piperidine **123** followed by transmetallation and Negishi cross-coupling with vinyl iodide **122**, which was followed by Boc-deprotection to yield styrene **124**. Hydrogenation of the *exo*-methylene moiety with Crabtree’s catalyst (nitrogen-directed chelation) was followed by oxidative carbonylative C–H lactamization to close the E-ring and furnish lactam **125**. The introduced lactam moiety was exploited as a directing group for *ortho*-C–H functionalization, introducing the aryl iodide functional handle for another cross-coupling. Afterwards, reduction of the lactam afforded the tricyclic DEF-fragment **126**.

Carbonylative cross-coupling of **121** and **126** proceeded in 1,4-dioxane at 60 °C to furnish dienone **127** in 73% yield. After exploring various acidic and photochemical conditions for the Nazarov cyclization of **127**, a combination of acetic acid and irradiation at 370 nm was found to be optimal. This was followed by in situ epimerization of C9–H with base to give *trans*-hydrindanone **118**. It was proposed by the authors, that the bulky C6–OTBS protecting group exerts a high degree of stereochemical control at C8 in the Nazarov cyclization. The undesired C9-epimer was also obtained in the reaction and could be epimerized to **118** by treatment with base.

From **118**, defunctionalization at C11 and deprotection were needed to reach heilonine (**16**). Two possible reaction sequences were disclosed by the authors: A two-step sequence of Clemmensen reduction and TBS-deprotection with TfOH afforded heilonine in 24% yield over 2 steps. An alternative sequence consisted of reduction of the C11-ketone to the secondary alcohol, followed by xanthate formation and Barton–McCombie deoxygenation and TBS-deprotection with TfOH (4 steps, 55%). This resulted in an overall longer sequence while improving the isolated yield of (+)-**16**. In total, heilonine was synthesized in 11 or 13 steps LLS from (*S*)-Wieland–Miescher ketone (**34**) [28].

Besides the two key steps building the D-ring (carbonylative cross-coupling and Nazarov cyclization), four C–H functionalization transformations were employed to enable the efficient total synthesis of (+)-**16**. These include the Na_2_-eosin Y-catalyzed visible-light-induced C–H hydroxylation, a Boc-directed C–H lithiation–Negishi cross-coupling, palladium-catalyzed carbonylative C–H lactamization, and lactam-directed rhodium-catalyzed C–H iodination.

#### Zygadenine

Zygadenine (**18**) constitutes the core alkamine for various natural products like veratroylzygadenine, vanilloylzygadenine, or zygacine. All are alkaloids occurring in death camas (*Zigadenus* spp.), a poisonous plant native to North America, which is often responsible for poisonings of livestock [54].

**Scheme 36:**
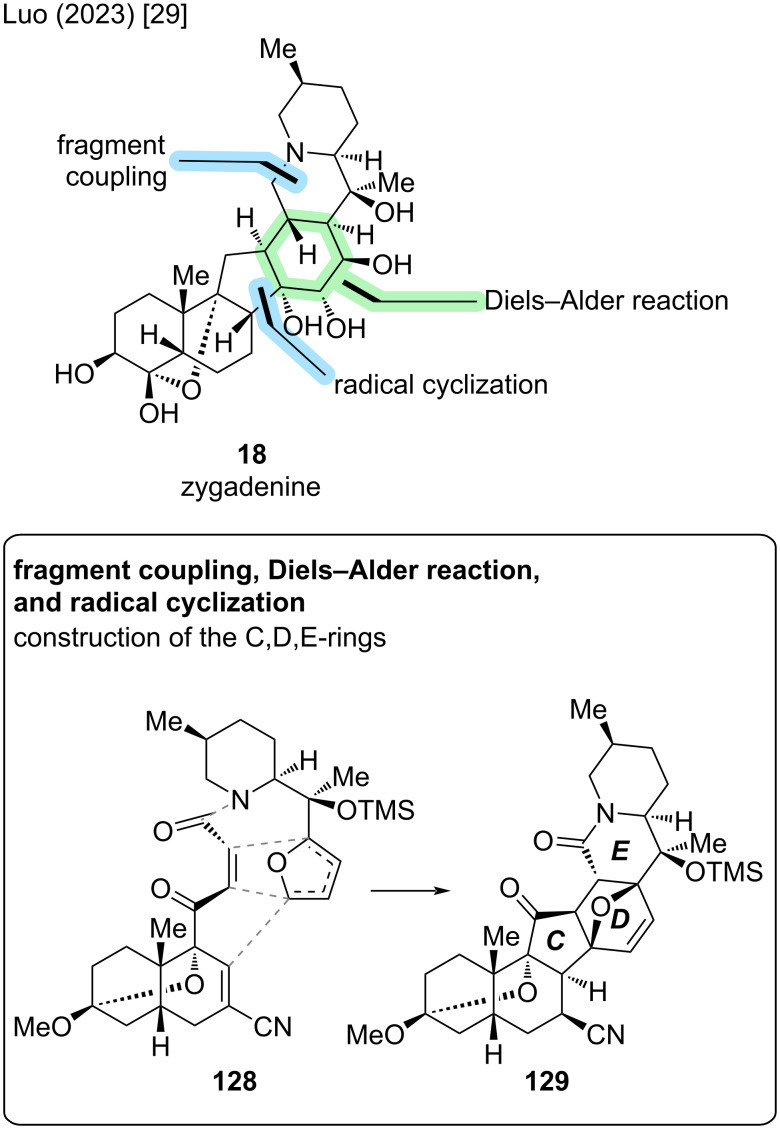
Key steps of the total synthesis of zygadenine (**18**) reported by Luo and co-workers [29].

In zygadenine (**18**), the C9–OH is connected to C4 to form a hemiacetal moiety. This structural feature is also present in other highly oxidized cevanine-type alkaloids like germine (**20**) and has posed challenges for total synthesis efforts (see 4-methylenegermine, Stork) [22]. Zygadenine (**18**) exhibits 15 stereocenters, 14 of which are contiguous. The first total synthesis of (−)-**18** was reported by Luo and co-workers in 2023 [29]. Key disconnections in their synthetic approach were an intramolecular Diels–Alder cycloaddition with furan and a radical cyclization to construct the C,D-rings (see Scheme 36). To set the stage for the key steps, a convergent coupling of a bicyclic dienophile fragment **137** and a furan-containing F-ring fragment **134** was devised, leading to connections indicated in **128** (Scheme 36).

**Scheme 37:**
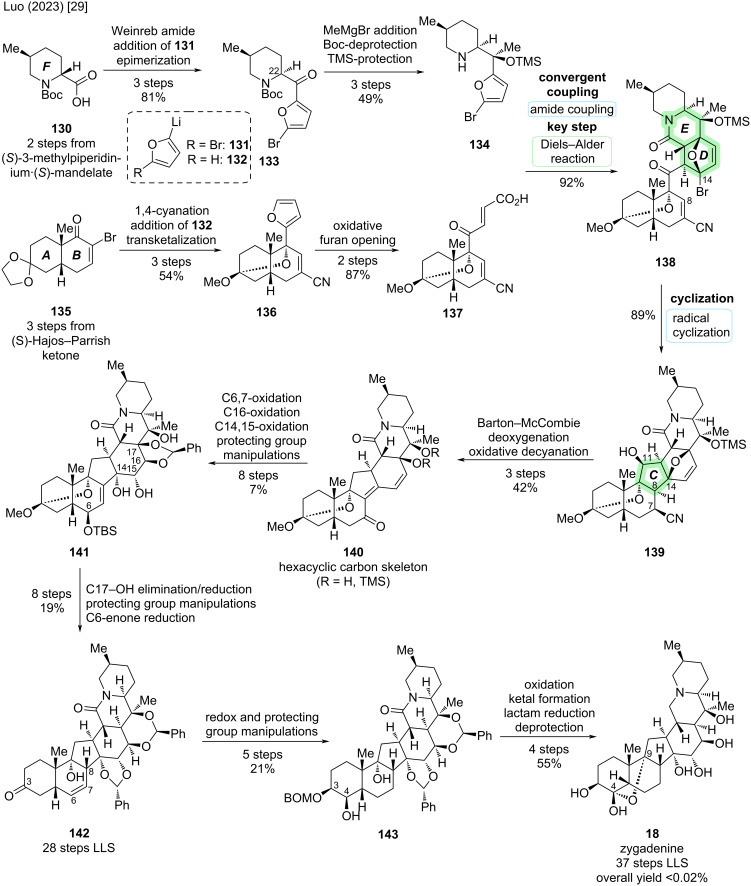
Overview of the total synthesis of zygadenine (**18**) by Luo and co-workers (2023) [29].

The synthesis of furan fragment **134** commenced with chiral piperidine-carboxylic acid **130** (2 steps from (*S*)-3-methylpiperidinium·(*S*)-mandelate, see Scheme 37) [55]. Weinreb amide formation was followed by the addition of 5-bromo-2-furanyllithium (**131**) and epimerization of C22 with DBU to give ketone **133**. Addition of MeMgBr to the ketone was guided by Felkin–Anh control to form a tertiary alcohol with the desired configuration. Boc-deprotection and TMS-protection of the tertiary alcohol furnished F-ring fragment **134**.

The synthesis of the AB-fragment **137** commenced from bicyclic enone **135**, which in turn was derived from (+)-Hajos–Parrish ketone in three steps, including a ring expansion [56]. 1,4-Cyanide addition and elimination were followed by a facial-selective 1,2-addition of 2-furanyllithium (**132**) to the ketone moiety. Treatment with CSA and MeOH induced a transketalization to **136** with an oxo bridge between C9 and C3. Notably, this is not the position of the ketal in the natural product and was rather employed as a masking group. To conclude the synthesis of dienophile **137**, the furan moiety of **136** was oxidatively opened with singlet oxygen, followed by *cis*–*trans*-isomerization of the double bond in the same pot and Pinnick–Lindgren oxidation.

Coupling of the secondary amine of **134** and the carboxylic acid moiety of **137** was immediately followed by heating to reflux in THF to afford the intramolecular Diels–Alder reaction, giving **138** as a single isomer in 92% yield and building the D- and E-rings. Formation of the C14–C8 bond to construct the C-ring was realized via a Giese-type radical addition of the radical generated from the C14-bromide to the C8-olefin. The C11-ketone was reduced in the same pot with NaBH_4_ to afford alcohol **139**. Notably, the radical cyclization resulted in the undesired epimer at C8, which needed to be corrected later in the synthetic sequence.

The obtained alcohol **139** was subjected to Barton–McCombie deoxygenation. Oxidative decyanation at C7 was accompanied by basic cleavage of the bridging oxygen in the D-ring to give dienones **140** as inconsequential regioisomers of the TMS protecting group (C17 or C20). At this stage, the synthesis of the hexacyclic carbon skeleton of zygadenine (**18**) was concluded.

Various oxidative transformations were employed to introduce oxidation at C6 (via silyl enol ether and dihydroxylation) and to equip the D-ring with appropriate hydroxy groups at C14, C15, and C16. This was complemented by manipulations of the protecting groups to ensure the desired reactivity. In intermediate **141**, all carbons bearing oxygen substituents in zygadenine (**18**) had now been oxidized. However, there was also extraneous degree of oxidation at C6, C17, and C18. In an eight-step sequence, defunctionalization at C17 (via elimination of C17–OH and hydrogenation) and at C6 (via deprotection/oxidation to enone/reduction) was followed by acetal cleavage to obtain 6,7-olefin **142**. Notably, this enone reduction now installed the correct configuration at C8. The 6,7-olefin and C3-carbonyl were reduced and thus-obtained C3–OH was eliminated. Dihydroxylation of the 3,4-olefin was followed by BOM-protection at C3 to yield **143**. To conclude the synthesis, C4–OH was oxidized to the ketone and the C4,9-hemiketal was installed, which was followed by Ir-catalyzed silane reduction of the lactam moiety and global deprotection. This concluded the de novo synthesis of (−)-zygadenine (**18**) in 37 steps LLS from (+)-Hajos–Parrish ketone with an overall yield of less than 0.02% [29].

According to the authors, the route offers potential for further optimization, for example by prefunctionalization of the A ring or identification of a new way to secure the correct C8 stereogenic center earlier in the synthetic sequence [29].

### Divergent syntheses of cevanine-type alkaloids

**Scheme 38:**
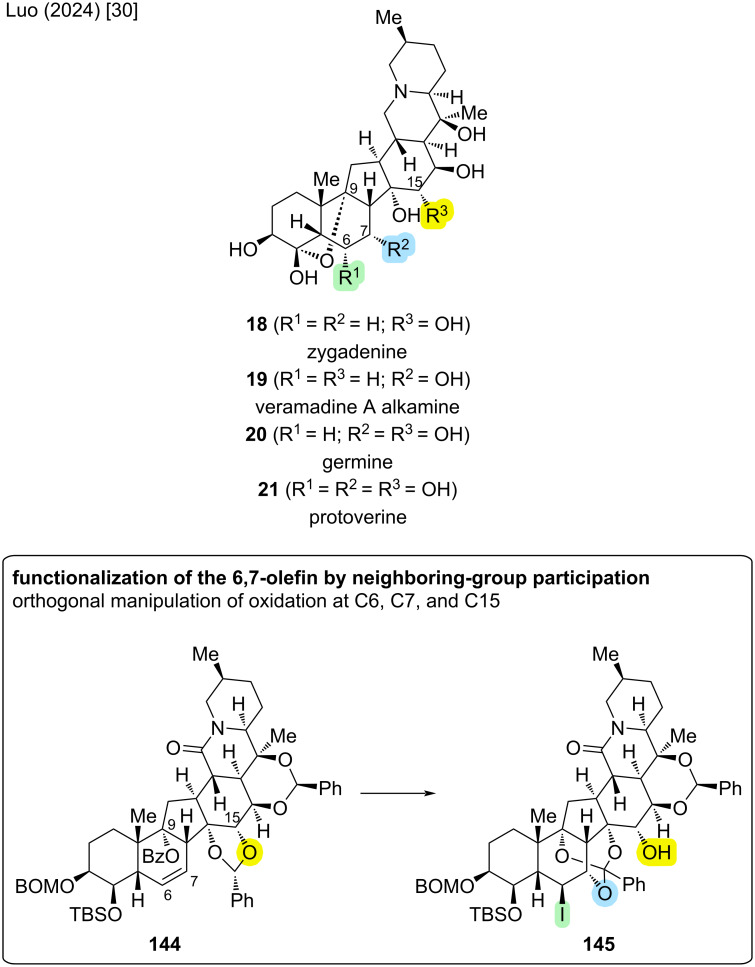
Key step of the divergent total syntheses of highly oxidized cevanine-type alkaloids by Luo and co-workers (2024) [30].

The so far most comprehensive effort toward the total synthesis of cevanine-type alkaloids was reported by Luo and co-workers (2024) [30]. Building upon their previously reported total synthesis of (−)-zygadenine (**18**) [29] and using an advanced intermediate from this publication, they achieved the divergent total syntheses of veramadine A alkamine (**19**), germine (**20**), and protoverine (**21**). Furthermore, a different route to late-stage intermediate **143** was disclosed, constituting a formal total synthesis of zygadenine (**18**). Zygadenine (**18**), germine (**20**) and protoverine (**21**) are distinguished by increasing oxidation levels, namely at C6, C7, and C15. Veramadine A has a veratroyl ester-side chain at C3–OH. The alkamine **19** displays the same oxidation level as **18**, although with oxidation at C7 instead of C15. To realize divergent total syntheses, Luo and co-workers devised an intermediate that allowed for orthogonal manipulation of oxidation levels at C6, C7, and C15. A key step toward enabling this divergent approach was the functionalization of the 6,7-olefin using neighboring group participation of the Bz-protected C9–OH (see Scheme 38).

**Scheme 39:**
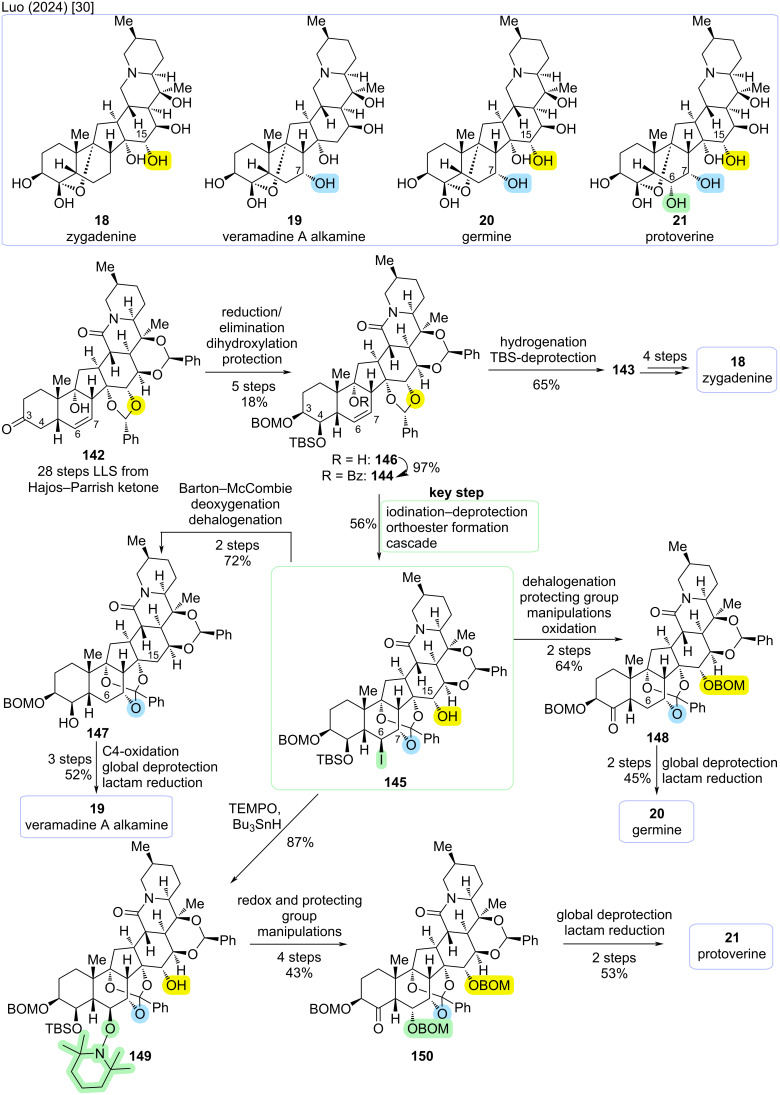
Divergent syntheses of highly oxidized cevanine-type alkaloids by Luo and co-workers (2024) [30].

The reported synthesis (see Scheme 39) commences from ketone **142** (28 steps LLS from (+)-Hajos–Parrish ketone), which was an intermediate in the previously communicated total synthesis of (−)-zygadenine (**18**) [29]. The hexacyclic carbon skeleton shared by all cevanine-type alkaloids was already constructed in this intermediate; only the installation of the correct oxidation pattern was required. In a five-step sequence, the C3–OH was converted to C3,4-diol **146** with orthogonal protecting groups, which served as one point of divergence. Preliminary studies indicated that the elimination of C3–OH and dihydroxylation of the obtained 3,4-alkene must occur before the neighboring-group participation reaction due to the required geometry for *trans*-elimination. Hydrogenation of the 6,7-double bond and TBS-deprotection afforded intermediate **143** which had been previously described in the total synthesis of zygadenine (**18**), offering an alternative pathway from **142** to **18** with a later point of divergence toward other cevanine-type alkaloids [30].

The C9–OH was benzoylated to furnish **144**. This set the stage for oxidation of the 6,7-double bond using neighboring group participation of the C9–OBz. Upon treatment with IBr, the iodonium species was intercepted in an intramolecular fashion by the C9–benzoyl group, which in turn reacted with the 14,15-benzylidene acetal to give orthoester **145**. This reaction not only installed an alcohol moiety at C7 with correct configuration, but also an iodide at C6. Furthermore, the C15–OH was now unprotected. Thus, **145** allowed for flexible adjustment of oxidation levels at C6 and C15 by functional group interconversions or defunctionalization, which are the discriminating positions between veramadine A alkamine (**19**), germine (**20**), and protoverine (**21**) [30].

To reach veramadine A alkamine (**19**) from **145**, C6 and C15 had to be defunctionalized. This was realized by converting the C15–OH to the corresponding xanthate ester. Treatment with AIBN/Bu_3_SnH afforded both the Barton−McCombie deoxygenation of C15–OH and reductive cleavage of the C6-iodide. In the same pot, this was followed by TBS-deprotection to furnish **147**. What remained was oxidation of the C4–OH, followed by global deprotection through hydrogenation with concomitant C4/C9-acetal formation and final reduction of the lactam. This concluded the first reported total synthesis of veramadine A alkamine (**19**) (12 steps from **142**; 40 steps LLS from Hajos–Parrish ketone, overall yield <0.01%) [30].

For germine (**20**), the C15–OH of **145** should be conserved, only the oxidation at C6 needed to be removed. Radical reduction of the C6-iodide was followed by protecting group manipulations in a one-pot fashion and subsequent DMP oxidation to give ketone **148** over 2 steps. By employing global deprotection and lactam reduction as previously, the total synthesis of germine (**20**) could be completed (11 steps from **142**; 39 steps LLS from Hajos–Parrish ketone, overall yield <0.01%) [30].

For the total synthesis of protoverine (**21**), the oxidation pattern at the B,D-rings in **145** should be conserved. First, the C6-iodide was exchanged for an OTEMPO substituent under radical conditions with retention of configuration to yield **149**. In a four-step sequence of protecting group- and redox manipulations, C4 was oxidized to the ketone and the configuration of C6–OH was inverted, yielding **150**. As previously, the last two steps consisted of global deprotection and lactam reduction to complete the first total synthesis of protoverine (**21**) (14 steps from **142**; 42 steps LLS from (+)-Hajos–Parrish ketone, overall yield <0.01%) [30].

### Comparison of strategies for the cevanine-type

To date, six distinct cevanine-type alkaloids have been synthesized: verticine (**15**) [46], heilonine (**16**) [24,28], zygadenine (**18**) [29–30], veramadine A alkamine (**19**) [30], germine (**20**) [30], and protoverine (**21**) [30]. Furthermore, the unnatural congener 4-methylenegermine (**17**) was synthesized in a racemic fashion [22]. Of these, only **15** was accessed in a semisynthesis via rearrangement of a steroid precursor to the C-*nor*-D-*homo*-framework [46]. The remaining natural products were accessed through total synthesis efforts with de novo construction of the hallmark hexacyclic framework. Of the successfully synthesized targets, only heilonine (**16**) was synthesized more than once [24,28]. Due to the structural diversity and varying complexity of cevanine-type alkaloids, a direct comparison of different total syntheses is difficult.

**Scheme 40:**
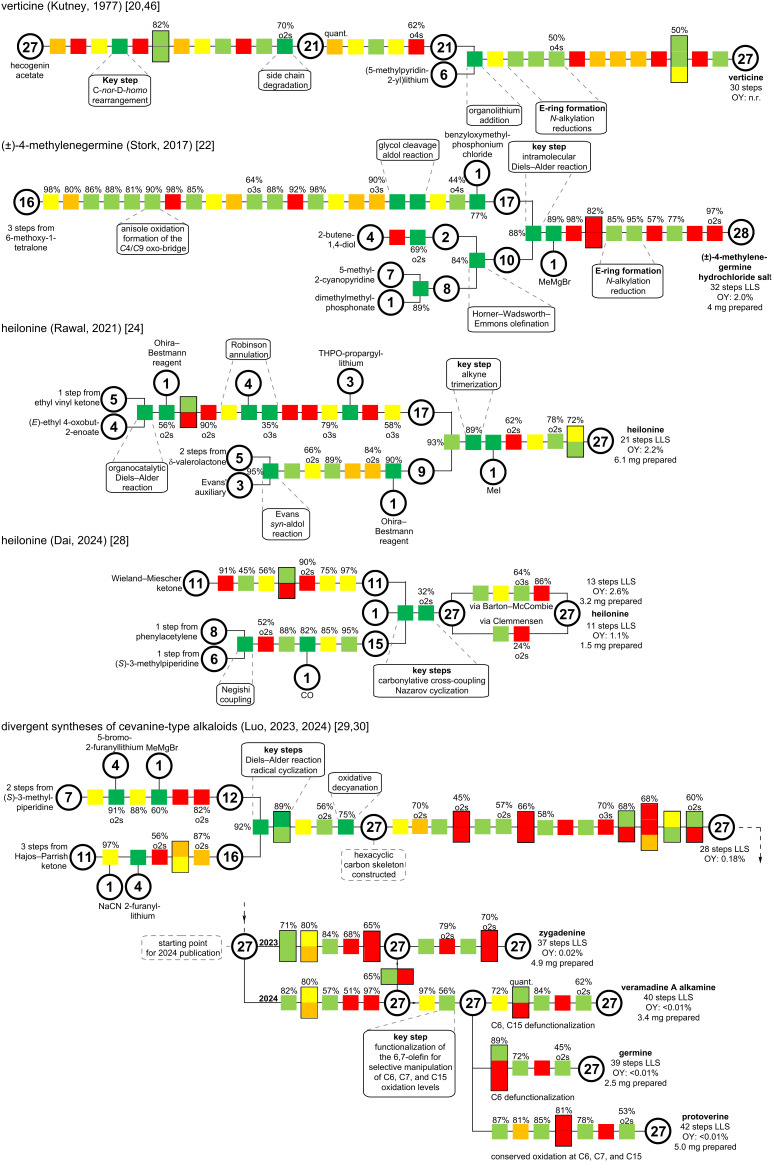
Color-coded overview of the presented cevanine-type alkaloid syntheses [10,20,22,24,28–30,46]. LLS: longest linear sequence; OY: overall yield.

A color-coded overview of the syntheses in the cevanine subgroup (Scheme 40) was created according to the method proposed by Schwan and Christmann (see the introduction for more information on the color-coding and definitions) [10].

As a result, Kutney’s semisynthesis of verticine (**15**) [20,46] is almost completely linear and comprises mainly redox manipulations. The only branch in the synthetic tree is the introduction of the F-ring through a pyridinyllithium species. Stork’s synthesis of (±)-4-methylenegermine (**17**) [22] features a convergent coupling of two fragments in a Diels–Alder reaction as the key step. The ABC-fragment is constructed in a rather long, linear sequence with several redox manipulations, contributing to prolonging the synthetic sequence.

Rawal’s total synthesis of heilonine (**16**) [24] featured a unique intramolecular alkyne trimerization to construct the C,D,E-rings in a selective and efficient fashion. The triyne precursor was obtained by convergent coupling of two fragments. Dai’s total synthesis of heilonine (**16**) [28] represents the most convergent and concise total synthesis of a cevanine-type alkaloid reported to date. This was achieved by exploiting four C–H-functionalization transformations, which enabled a convergent, carbonylative cross-coupling of two carefully prefunctionalized fragments, and a Nazarov cyclization to construct the C-ring.

The divergent syntheses of zygadenine (**18**), veramadine A alkamine (**19**), germine (**20**), and protoverine (**21**) disclosed by Luo and co-workers [29–30] are collectively represented in a network to highlight the divergent nature of the synthetic blueprint. Compared to previous syntheses, the step count and overall efficiency are inferior. However, when considering the complexity of the targets and the divergent access to them, this becomes secondary. The construction of the hexacyclic carbon framework was completed after 13 steps in a convergent fashion, which compares well with previous total synthesis efforts. The extensive redox and protecting group manipulations required to install the complex oxidation pattern resulted in a long, linear reaction sequence. It remains to be seen how these and other sequences can be shortened either by more selective redox transformations or by prefunctionalization of the fragments before convergent coupling.

## Conclusion

In this review, we aimed to give a comprehensive overview of synthetic strategies toward the *Veratrum* alkaloids. Visual aid in comparison of strategies in the subclasses of jervanine-, veratramine-, and cevanine-type *Veratrum* alkaloids is provided by the color-coded Christmann schemes (Schemes 15, 26, and 40) [10].

A rise in synthetic approaches was observed commencing from the first synthesis of cyclopamine in 2009 by the Giannis group [21], with the Liu/Qin semisynthesis of 2024 [26] being the shortest known synthesis through a similar biomimetic rearrangement. Discussed were also two different convergent approaches: one cut through the D-ring via a key Tsuji–Trost and RCM sequence [23], and another cut through the C-ring featuring a Nazarov cyclization [25].

Careful observations in the total syntheses of veratramine showed different synthetic cuts enabling sequences comparable in overall step count. Methods included a modified Wagner–Meerwein-rearrangement in a divergent semisynthesis [26], the aforementioned Nazarov cyclization [25], where veratramine provided a relay synthesis toward cyclopamine, and a one-pot Eschenmoser fragmentation/HWE/methylation sequence followed by a Diels–Alder/aromatization cascade [27].

A unifying feature of all cevanine-type total synthesis efforts is the convergent coupling of two fragments of similar size, either accompanied or directly followed by the construction of one or both of the central C- and D-rings. Methods for the construction of the 6-membered D-ring included Diels–Alder reactions [22,29] or alkyne trimerization [24], while the 5-membered C-ring was constructed using the Nazarov reaction [28], radical cyclization [29], or aldol condensation [49]. Another common disconnection was the construction of the C18–N bond in the E-ring by alkylation or amide coupling. The piperidine moiety in the F-ring was either introduced directly using a chiral 3-methylpiperidine building block or by reduction of the corresponding pyridine. The introduction of the A-B-ring was typically achieved using strategies and intermediates from conventional steroid total synthesis (Robinson annulation, Wieland–Miescher ketone, Hajos–Parrish ketone, etc.).

A general observation is the increasing complexity of successfully synthesized targets following a chronological order. This highlights the correlation of novel methods developed and the complexity of natural products accessible by total synthesis. A wide range of strategic C–C-bond forming reactions have been exploited to construct the different frameworks of *Veratrum* alkaloids in an elegant fashion. This was complemented by carefully orchestrated protecting group manipulations to install the complex hydroxylation pattern in the cevanine-type alkaloids. It is noteworthy that the oxidation at C12, as present for example in veracevine (**86**), has not yet been addressed, highlighting a further possibility for innovation. It is our expectation that the coming years will see more elegant and concise approaches to the synthesis of these complex natural products.

## Data Availability

Data sharing is not applicable as no new data was generated or analyzed in this study.
